# Using a Semi-Automated Strategy to Develop Multi-Compartment Models That Predict Biophysical Properties of Interneuron-Specific 3 (IS3) Cells in Hippocampus

**DOI:** 10.1523/ENEURO.0087-16.2016

**Published:** 2016-09-19

**Authors:** Alexandre Guet-McCreight, Olivier Camiré, Lisa Topolnik, Frances K. Skinner

**Affiliations:** 1 Krembil Research Institute, University Health Network, Toronto, Ontario, M5T 2S8, Canada; 2 Department of Physiology, University of Toronto, Toronto, Ontario, M5S 1A8, Canada; 3 Department of Biochemistry, Microbiology and Bio-informatics, Neuroscience Axis, CHU de Québec Research Center (CHUL), Laval University, Québec City, Québec, G1V 0A6, Canada; 4 Departments of Medicine (Neurology) and Physiology, University of Toronto, Toronto, Ontario, M5S 1A8, Canada

**Keywords:** hippocampus, interneuron, modeling

## Abstract

Determining how intrinsic cellular properties govern and modulate neuronal input–output processing is a critical endeavor for understanding microcircuit functions in the brain. However, lack of cellular specifics and nonlinear interactions prevent experiments alone from achieving this. Building and using cellular models is essential in these efforts. We focus on uncovering the intrinsic properties of *mus musculus* hippocampal type 3 interneuron-specific (IS3) cells, a cell type that makes GABAergic synapses onto specific interneuron types, but not pyramidal cells. While IS3 cell morphology and synaptic output have been examined, their voltage-gated ion channel profile and distribution remain unknown. We combined whole-cell patch-clamp recordings and two-photon dendritic calcium imaging to examine IS3 cell membrane and dendritic properties. Using these data as a target reference, we developed a semi-automated strategy to obtain multi-compartment models for a cell type with unknown intrinsic properties. Our approach is based on generating populations of models to capture determined features of the experimental data, each of which possesses unique combinations of channel types and conductance values. From these populations, we chose models that most closely resembled the experimental data. We used these models to examine the impact of specific ion channel combinations on spike generation. Our models predict that fast delayed rectifier currents should be present in soma and proximal dendrites, and this is confirmed using immunohistochemistry. Further, without A-type potassium currents in the dendrites, spike generation is facilitated at more distal synaptic input locations. Our models will help to determine the functional role of IS3 cells in hippocampal microcircuits.

## Significance Statement

For any given neuron, its intrinsic properties determine the conversion of synaptic inputs into spike output. Nonetheless, the intrinsic profile of many neuronal types remains largely unknown due to the absence of cell-specific tools and technical challenges. To overcome this, we developed multi-compartment models to make predictions about cellular intrinsic properties and input–output relationships. We used a semi-automated strategy involving populations of models to capture electrophysiological features of the cell type. We focused on type 3 interneuron-specific cells, a class of GABAergic interneurons that may exert disinhibitory control on hippocampal microcircuits. Our models predicted the presence of fast delayed rectifier potassium currents and the absence of slow delayed rectifier channels, and this was confirmed experimentally.

## Introduction

Uncovering the intrinsic cellular properties that control the input–output processing within brain microcircuits is highly challenging due to the large diversity of voltage-gated ion channels (VGCs) expressed by individual neurons and their variable distribution across the cell structure. In particular, the spatial location of VGCs determines the integration of synaptic inputs by specific subcellular regions. Because of the difficulty in investigating subcellular properties with purely experimental means, multi-compartmental modeling has been used as a key tool to explore the contributions of different VGC types (Carnevale and Hines, 2006).

Traditionally, morphologically detailed multi-compartment models are developed using hand-tuning methods (De Schutter and Bower, 1994; Migliore et al., 1995, 1999; Saraga et al., 2003; Lawrence et al., 2006; Hu et al., 2010), where passive and active parameter values are adjusted manually until the model reproduces experimentally observed electrophysiology characteristics (e.g., spike frequency). Recognizing that similar outputs can be achieved with different sets of parameter values, along with inherent biological variability, model database approaches have been developed (Prinz et al., 2003; Günay et al., 2008; Hay et al., 2011; Sekulić et al., 2014). These approaches typically generate populations of models by varying VGC maximal conductances and extracting a subset of the models that best match the experimental data. Often, however, information on the types of VGCs and their biophysical details is not available. While having multi-compartment models to determine the roles of particular cell types in microcircuit dynamics is useful, how best to proceed in building multi-compartment models with limited experimental data is unclear. Furthermore, some sort of cyclic approach between model and experiment is clearly warranted, as models require continuous updating as more experimental data becomes available. In particular, Sekulić et al. (2014) designed a cyclic model database approach where the database design hinged on a particular question. In this way, they not only obtained populations of models representing the particular cell type for subsequent examination, but they also motivated specific experimental studies for future consideration. However, their starting point requires that a “reference” multi-compartment model with known densities and distributions of VGCs that can capture the experimental data already exists. This would typically have arisen from previous hand-tuning work. Given that not all cell types possess such multi-compartment model representations, and that hand tuning is not the most efficient method for investigating parameter spaces, developing other approaches would be useful.

Here, we devised a strategy that uses populations of models in conjunction with hand tuning to generate multi-compartment models with characteristics that match features from electrophysiological recordings. We chose not to fully automate the process to allow flexibility in the model-building process, when no particular VGC data are available. At the same time, the partial automation through use of populations of models reduced the subjectivity and tediousness of pure hand tuning. We applied our strategy to obtain reference multi-compartment models for an identified cell type, the CA1 type 3 interneuron-specific (IS3) cell in the hippocampus. The models provided suggestions of types, densities and distributions of VGCs in IS3 cells.

The IS3 cell is a type of inhibitory interneuron that exclusively targets other interneurons and does not contact pyramidal cells (Acsády et al., 1996a,b; Gulyás et al., 1996). IS3 cells may receive excitatory inputs from the entorhinal cortex via the perforant path, from the CA3 area via Schaffer collaterals (SCs) and from CA1 local collaterals (Francavilla et al., 2015). Through photostimulation experiments and paired recordings, it has been shown that IS3 cells provide a major local source of inhibition to oriens-lacunosum moleculare (OLM) cells, and optimally control their firing at theta frequencies (Chamberland et al., 2010; Tyan et al., 2014). OLM cells in turn are known to provide a gating function in the information flow from the perforant path to the hippocampus (Maccaferri and McBain, 1995; Leão et al., 2012). We used our developed multi-compartment models with different combinations of VGCs to investigate how the type and location of dendritic conductances in conjunction with synaptic input influenced the IS3 output.

Our strategy for building multi-compartment models can be considered for other cell types where no VGC information is available. The developed models can then provide guidance to experimentalists in narrowing down which biological details are both plausible and important in microcircuit function. Moreover, the models can be used as reference models for subsequent examination. In this study, our developed multi-compartment IS3 cell models with fast delayed rectifier potassium channels distributed in the soma (S) and proximal dendrites (D) can capture the experimental data.

## Materials and Methods

### Slice preparation

Transverse hippocampal slices (300 μm) were prepared from the vasoactive intestinal polypeptide/enhanced green fluorescent protein (VIP-eGFP; Gensat) mice of either sex (postnatal day 14–23) in accordance with the animal welfare guidelines of Laval University. Animals were anesthetized deeply with isoflurane and decapitated. The brain was dissected carefully and transferred rapidly into an ice-cold (0 to +4°C) solution containing the following (in mm): 250 sucrose, 2 KCl, 1.25 NaH_2_PO_4_, 26 NaHCO_3_, 7 MgSO_4_, 0.5 CaCl_2_, and 10 glucose oxygenated continuously with 95% O_2_ and 5% CO_2_, pH 7.4, 330–340 mOsm. Slices were cut using a vibratome (Microm, Fisher Scientific); were transferred to a heated (35°C) oxygenated recovery solution containing the following (in mm): 124 NaCl, 2.5 KCl, 1.25 NaH_2_PO_4_, 26 NaHCO_3_, 3 MgSO_4_, 1 CaCl_2_, and 10 glucose; pH 7.4, 300 mOsm; and were allowed to recover for 45 min. Subsequently, samples were kept at room temperature until use.

### Electrophysiological recordings

Slices were perfused with standard artificial CSF containing (in mm): 124 NaCl, 2.5 KCl, 1.25 NaH_2_PO_4_, 26 NaHCO_3_, 2 MgSO_4_, 2 CaCl_2_, and 10 glucose saturated with 95% O_2_ and 5% CO_2_, pH 7.4, at 28–32°C. VIP-eGFP cells were visually identified for whole-cell patch-clamp recordings. For current-clamp recordings, the intracellular solution contained (in mm): 130 KMeSO_4_, 2 MgCl_2_, 10 diNa-phosphocreatine, 10 HEPES, 2 ATP-Tris, 0.2 GTP-Tris, and 0.3% biocytin (Sigma-Aldrich), pH 7.2–7.3, 280–290 mOsm/L. To block synaptic activity in some recordings, gabazine (2 μm) as well as glutamate receptor antagonists l-AP5 (50 μm) and NBQX (12.5 μm) were applied. For hyperpolarization-evoked responses (i.e., −100 and −90 pA current injections), 25 traces were taken from 14 different cells. For passive depolarization-evoked responses (i.e., 10 and 20 pA current injections), 11 traces were taken from eight cells. For active depolarization-evoked responses (i.e., 10–140 pA current injection), 56 traces were taken from 14 cells. For depolarization-evoked spike-block recordings (i.e., 450–700 pA current injections), 18 traces were taken from three cells.

### Two-photon calcium imaging

**Figure 1.**
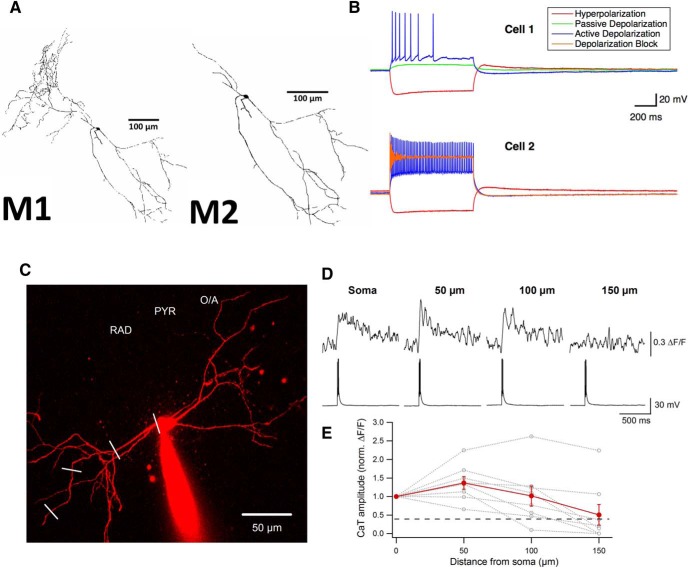
Morphological, membrane, and dendritic properties of IS3 cells. ***A***, Topological model of an IS3 cell full morphology (M1) and with axonal branches removed (M2). ***B***, Experimental IS3 cell traces during current-clamp recording showing IS3 cell electrophysiological features in two cells. In cell 1, current step injections show hyperpolarization with minimal sag (−100 pA; red), passive response without spiking (+10 pA; green), and depolarization with irregular spiking (+50 pA; blue). In cell 2, current injections show hyperpolarization with minimal sag (−100 pA; red), depolarization with spiking (+50 pA; blue), and depolarization block (+500 pA; orange). Recordings were obtained in the presence of synaptic blockers (i.e., NBQX, AP5, and gabazine). ***C***, Two-photon image of an IS3 cell filled with Alexa Fluor 594 and OGB-1. White lines indicate where backpropagating action potential-evoked Ca^2+^-transient (bAP-CaT) line scans were performed. The protocol consisted of three consecutive 2 ms, 800 pA somatic current step injections in conjunction with dendritic CaT recordings. ***D***, Representative examples of dendritic bAP-CaTs evoked by three APs at the soma. ***E***, Summary plot showing average changes (solid red) and individual changes (dotted gray) in the bAP-CaT amplitude at different distances from the soma. The dashed black line indicates the threshold level below which the calcium signal was indistinguishable from noise.

Dendritic Ca^2+^ imaging was performed using a TCS SP5 two-photon laser-scanning microscope (Leica Microsystems) based on a Ti-sapphire laser (Chameleon Ultra II, Coherent; >3 W, 140 fs pulses, 80 Hz repetition rate) tuned to 800 nm. A long-range water-immersion objective [40×; numerical aperture (NA), 0.8] was used to collect photons in the epifluorescence mode with external photomultiplier tubes. Neurons were filled with Oregon Green^®^ 488 BAPTA-1 (OGB-1) via the patch electrode for 20–30 min before imaging. Red fluorescence from Alexa Fluor 594 was used to locate dendrites of interest. To measure Ca^2+^ signals, green fluorescence was collected during 400 Hz line scans across dendritic segments of 2–15 μm. Fluorescence changes were quantified as changes in green fluorescence from the baseline (i.e., (*F* − *F*_0_)/*F*_0_), referred to as Δ*F*/*F* in Figure 1*C–E*.

### Morphological reconstructions

For morphological reconstructions of IS3 cells, recorded neurons were filled with biocytin (Sigma-Aldrich) during whole-cell recordings. Slices with recorded cells were fixed with 4% paraformaldehyde (PFA), rinsed in TBS, and incubated with Streptavidin-Alexa Fluor 488 overnight at +4^°^C, following which the slices were mounted. Confocal *z*-stacks were acquired using a Leica SP5 microscope (Leica Microsystems) with a 1 μm step. Morphological reconstructions were performed using the Neurolucida 8.26.2 software (MBF Bioscience).

### Neurochemical analysis

For neurochemical analysis, animals were perfused with 4% PFA and hippocampal sections (thickness, 30 μm) were prepared using a vibratome (VT1000, Leica Microsystems). Sections were permeabilized with 0.1% Triton X-100 in TBS and incubated in blocking solution containing 20% normal goat serum for 1 h. After this step, sections were incubated with anti-GFP (chicken, 1:1000; Aves Labs) and either anti-KCNB1 (mouse, 1:1000; Sigma-Aldrich) or anti-KCNC1 (rabbit, 1:500; Sigma-Aldrich) primary antibodies at room temperature for 24–48 h. For KCNC1, slices were incubated the following day with biotinylated anti-rabbit (goat, 1:200; Vector Laboratories) antibodies for 4 h at room temperature, then for 24 h with A/B reagents (1:100; Vectastain ABC Kit, Vector Laboratories) at room temperature. The following day, slices were incubated for 2 h with the following conjugated secondary antibodies: anti-chicken Alexa Fluor 488 (1:1000; Jackson ImmunoResearch); and either anti-streptavidin Alexa Fluor 546 (1:200; Jackson ImmunoResearch) or Alexa Fluor 647 (1:250; Invitrogen). Slices were then rinsed and mounted on microscope slides. Confocal images of labeled sections were obtained using a Leica TCS SP5 imaging system equipped with a 488 nm argon, a 543 nm HeNe, and a 633 nm HeNe laser, with a 63× (NA, 1.4) oil-immersion objective (Leica Microsystems). Based on previous observations (Tyan et al., 2014), GFP-expressing bipolar cells with a small size cell body (10–15 μm) and vertically oriented dendrites were considered as putative IS3 cells, and were used for analysis of the voltage-gated K^+^ channel subunit (Kv) expression.

### Simulation and analysis software

All simulations were performed using the NEURON software environment (Carnevale and Hines, 2006). In some instances, these simulations were computed using the Neuroscience Gateway for high-performance computing (Sivagnanam et al., 2013). All simulations as well as experimental recordings were analyzed using a MATLAB toolbox called PANDORA (Günay et al., 2009) as well as customized MATLAB code. Models will be made publicly available on ModelDB (Hines et al., 2004) following publication of this article.

### Model morphology and compartmentalization

Topological information provided by the morphological reconstruction of an IS3 cell was imported directly into NEURON. The morphological structure was then subdivided into multiple compartments until the point where the compartments were small enough to be considered isopotential. The topological model is shown in Figure 1*A*. Because inclusion of the full axon in the model requires more specifics regarding biophysical axonal properties (i.e., channels types and distributions) and more computational strain, we considered a reduced morphological version where the axon is cut down to a small remaining section (M2) relative to the full version with the axon intact (M1; Fig. 1*A*). In the M2 version, there were 221 compartments, compared with 653 in the full M1 version, and the surface area was reduced from 20,871 to 7297 μm^2^ (∼35% of the original surface area). While both morphologies were considered, we focused on the M2 version.

### Model passive properties

The passive parameters of the model were adjusted such that resting membrane potential, input resistance, and membrane time constant were matched with their appropriate experimentally observed values. The passive properties of the model were matched with raw data from the respective cell, and not with the experimental averages. The specific membrane capacitance and axial resistance parameters were restricted to appropriate experimentally estimated ranges. The average measurement of specific membrane capacitance in cortical pyramidal neurons, spinal cord neurons, and hippocampal neurons, including interneurons, is 0.9 μF/cm^2^ (Chitwood et al., 1999; Gentet et al., 2000), suggesting that the range for this parameter is fairly consistent across many neuron types. Values for axial resistance in individual neurons can range from 50 to 400 Ω/cm (Dayan and Abbott, 2005; Holmes, 2010).

Furthermore, in the case of the M2 morphology, passive parameter values in the soma and dendrites were kept the same as the passive parameter values in the M1 morphology, but the passive parameter values in the remaining axon sections were readjusted in order to get appropriate resting membrane potential, input resistance, and membrane time constant values. This adjustment was performed to compensate for the axonal surface area difference and is a strategy that has been used previously (Lawrence et al., 2006). Passive properties for M1, M2, and experimental data are given in Table 1.

**Table 1:** Model passive properties

Morphology	*R_N_* (MΩ)	*τ_m_* (ms)	Resting *V_m_* (mV)	*C_m_* (μF/cm^2^)	*R_a_* (Ω/cm)	*G_m_* (S/cm^2^)
M1	411.8	24.2	−69.7	0.9	255	0.000019
M2	414.3	24.2	−69.7	4 (Axon)	300 (axon)	0.000185 (axon)
Experimental/literature	413.0	24.2	−69.7	0.9 ± 0.3	100-300	N/A

Passive property measurements (columns 2–4) and passive parameter values (columns 5–7) for both computational morphologies (without active properties) compared with the experimental (columns 2–4) and literature (columns 5–7) values. Note that for M2, the parameter values listed refer to the values set for the remaining axon segments. The M2 somatic and dendritic parameter values are the same as those listed for M1. *R_N_*, input resistance; *τ_m_*, membrane time constant; *V_m_*, membrane potential; *C_m_*, specific membrane capacitance; *R_a_*, axial resistance; *G_m_*, specific membrane conductance; N/A, not applicable.

### Electrotonic analysis in passive model

Using the passive model and the Electrotonic Analysis toolbox in NEURON, we looked at the natural log of voltage attenuation across the morphology of the model, with the soma as the electrode reference point. In this case, the natural log of voltage attenuation is considered the electrotonic distance (i.e., *L* = log[*A*]), and attenuation (*A*) is measured as: *A* = voltage upstream/voltage downstream (i.e., where voltage upstream is an applied 1 mV signal and voltage downstream is the downstream response to the 1 mV signal). This definition of electrotonic distance allows for a direct relationship to attenuation, regardless of the cellular morphology. For this analysis, both voltage spreading toward the soma (*V*_in_) as well as away from the soma (*V*_out_) were plotted.

### Semi-automated strategy

**Figure 2.**
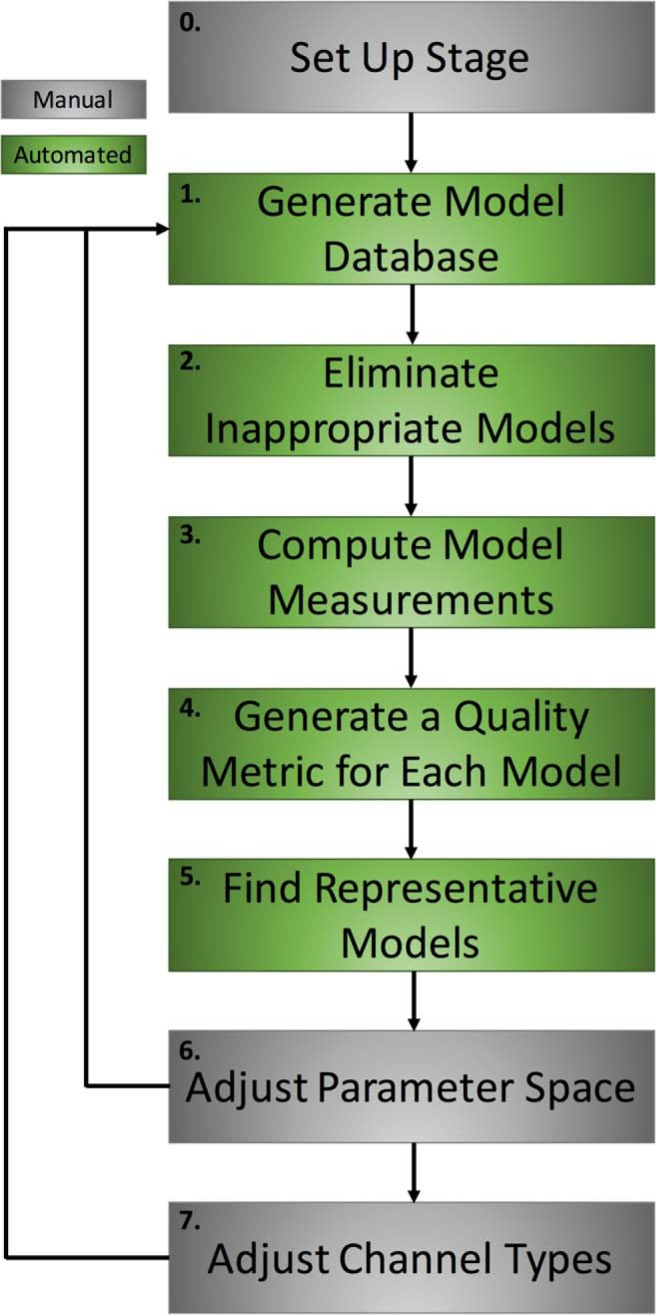
Semi-automated strategy. Flowchart outlining the steps in the approach that we used to develop the IS3 cell model databases. Note that gray boxes indicate steps that were performed manually through hand tuning, and green boxes indicate steps that were automated within NEURON and MATLAB.

**Figure 3.**
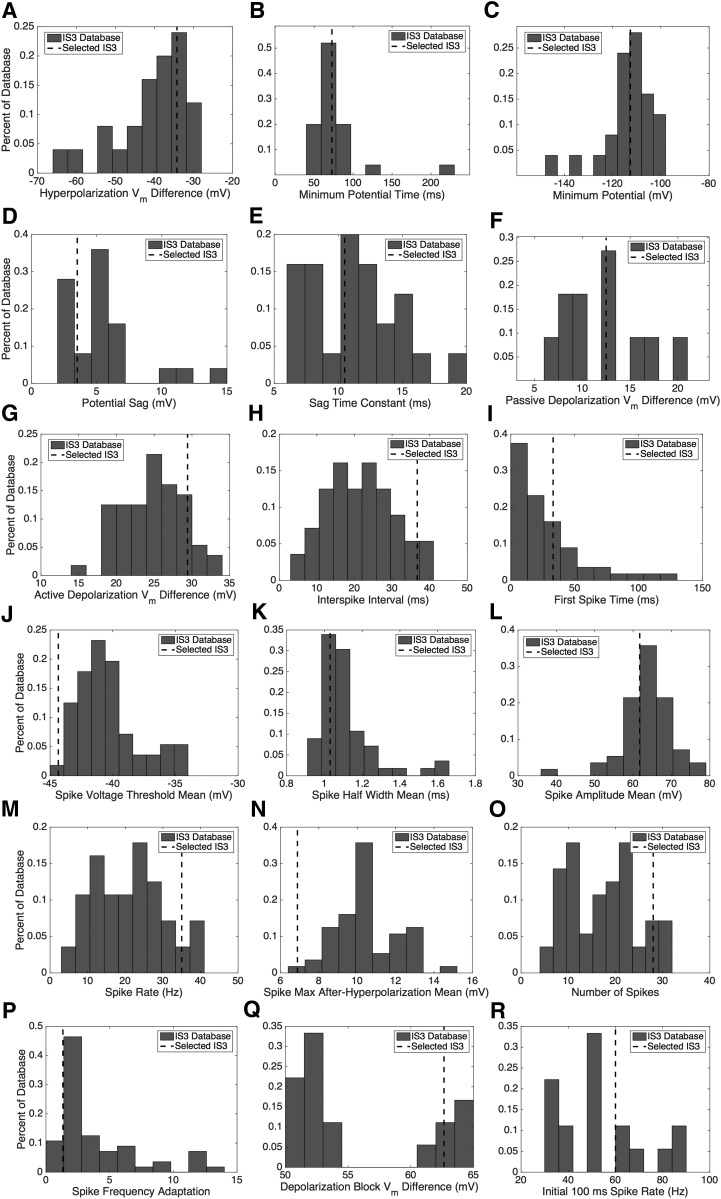
Experimental measurement histograms. ***A–R***, The histograms are generated from data where the criterion is that they exhibit hyperpolarization (***A–E***; CIPs include −100 and −90 pA for the “lack of sag feature”), passive depolarization (***F***; CIPs include 10 and 20 pA for the “no spiking feature”), depolarization with spiking (***G–P***; CIPs range from 10 to 140 pA with 10 pA intervals for the “normal spiking feature”), or depolarization block (***Q***, ***R***; CIPs range from 450 to 700 pA with 50 pA intervals for the “depolarization block feature”). Note that the dashed lines indicate the measurements obtained from the selected IS3 cell experimental trace used to compute the distance metric for each model. The signature features, and characteristic measurements and their values are given in Table 2 (second, third, and fourth columns).

**Figure 4.**
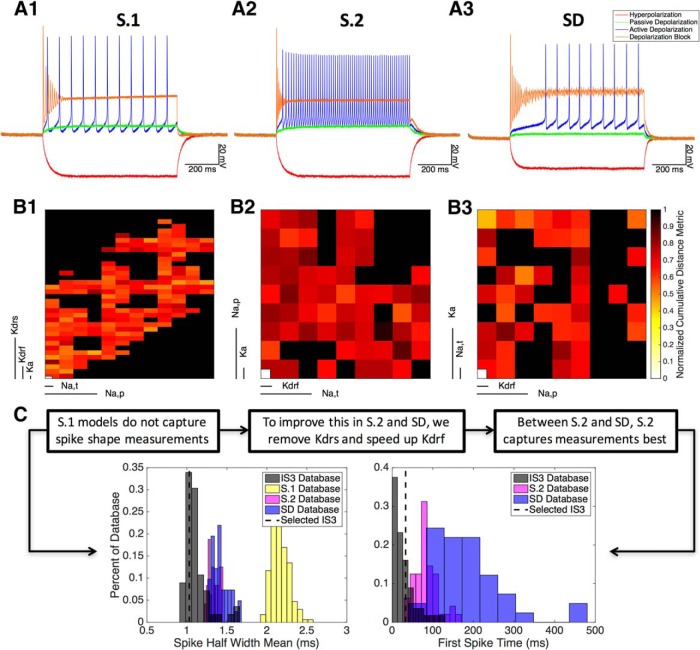
Semi-automated strategy and model databases. ***A1–A3***, Top models in S.1, S.2, and SD. The current injection protocol is −100, +20, +50, and +500 pA. ***B1–B3***, Parameter spaces for S.1, S.2, and SD (from left to right), as visualized using CBDR. Each pixel represents a single model and the distance metric of that model. Conductance axes are organized such that overall low-conductance models are in the bottom left quadrants and overall high-conductance models are in the top right quadrants. Black pixels represent models that are eliminated in step 2 and are assigned a distance value of 100 as a result. For S.1, S.2, and SD, respectively, 182 of 432, 59 of 81, and 42 of 81 models did not get rejected (i.e., the remaining colored pixels). ***C***, Example experimental and model database (i.e., for S.1, S.2, and SD) voltage trace measurement histograms for spike half-width and first spike time during 50 pA stimulation. The additional flowchart above the figures shows example rationales for the changes that were made among the S.1, S.2, and SD distributions, including reasoning for why the S.2 models best captured IS3 experimental features.

**Figure 5.**
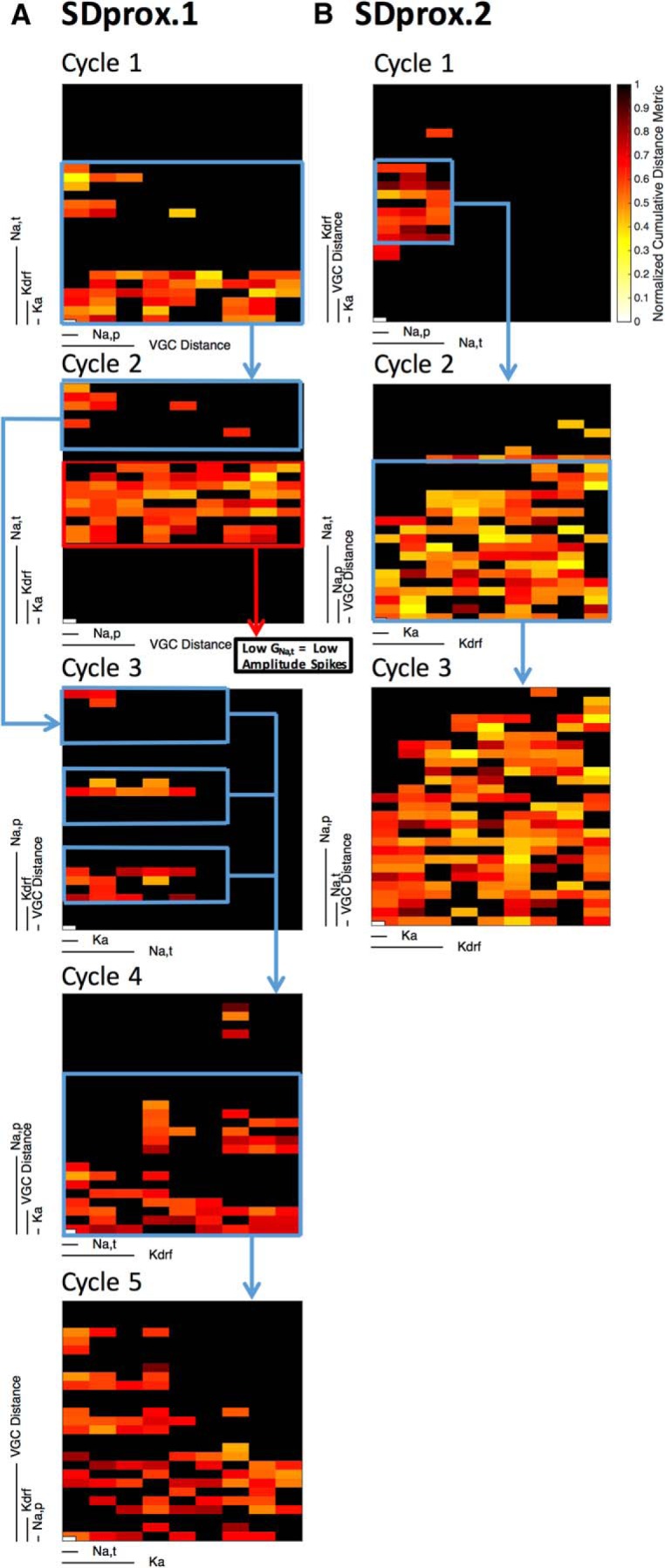
Example adjustments made in between parameter refinement cycles for SDprox.1 and SDprox.2 model databases. ***A***, For SDprox.1, five cycles of steps 1 to 6 were required (Cycle 1 = 47 remaining of 243 models; Cycle 2 = 64 remaining of 243 models; Cycle 3 = 22 remaining of 243 models; Cycle 4 = 52 remaining of 243 models; Cycle 5 = 70 remaining of 243 models). ***B***, for SDprox.2, three cycles of steps 1 to 6 were required (Cycle 1 = 24 remaining of 243 models; Cycle 2 = 107 remaining of 243 models; Cycle 3 = 146 remaining of 243 models). The CBDR plots show the quality of the parameter space of the model database during each cycle. Note that blue-boxed areas indicate parameter spaces of interest that were focused on. Red-boxed areas indicate parameter spaces that were purposely avoided.

To determine VGC types, densities, and distributions in IS3 cells, we used an approach that combined hand tuning with automation, allowing both automated simulations and examination of multiple features to simultaneously capture various modes of IS3 cell dynamics. The steps in this strategy (illustrated in Fig. 2) are outlined here, where each step is prefaced by whether it is an automated (Auto) or manual (Hand) step:

0. *[Hand] Setup stage:* a model morphology with passive properties needs to be in hand along with the chosen experimental electrophysiological signature features obtained at different current steps [i.e., current injection protocols (CIPs)]. The key signature features used for IS3 cells are a lack of sag during hyperpolarization, a passive response without spiking, depolarization with normal spiking, and depolarization block (Table 2, second column; Fig. 1*B*). Experimental traces with these features were selected and they had CIPs of −100, +20, +50, and +500 pA (Table 2, first column). Additionally, an initial set of VGC types needs to be chosen and hand tuning used to find a single set of parameter values that approximately matches electrophysiological features. These parameter values are then used as a basis to generate a population of models. We chose three to four parameter values that encompassed each of the initial hand-tuned values (the maximal conductance values were used; see Table 6).
1. *[Auto] Generate model database:* a population of models was generated in the NEURON software environment where each model possessed a unique combination of channel conductance values. The CIPs were applied to each model, and the voltage traces generated from each model were imported into MATLAB using PANDORA (Günay et al., 2009) and organized into databases.
2. *[Auto] Eliminate inappropriate models:* the models that did not capture IS3 cell features at the given CIPs were eliminated. The criteria used were based on an examination of the experimental data (see Results and Table 2, fifth column).
3. *[Auto] Compute model measurements:* for the remaining models, characteristic measurements for each feature were determined (Table 2, third column). Specifically, each CIP possessed its own set of characteristic measurements (e.g., spike amplitude mean at +50 pA, potential sag at −100 pA). For −100 pA, 5 measurements were evaluated; for +20 pA, 1 measurement was evaluated; for +50 pA, 10 measurements were evaluated; and for +500 pA, 2 measurements were evaluated. Although computation of most of these measurements already exists in the PANDORA toolbox, the following two customized measurements needed to be added: membrane potential (*V*_m_) difference and membrane potential difference in the last 700 ms of the CIP.
4. *[Auto] Generate a quality metric for each model:* each measurement for each model was compared with the same measurement from the selected experimental traces representing the signature feature. Dashed lines in Figure 3 histograms show the measurements obtained from the selected traces (Table 2, fourth column) used in comparison with the model measurements. The normalized Euclidean distance (Günay et al., 2009) between each model and selected experimental trace measurements was then computed as follows:
  $$d_{x,y}=\sum_{i=1}^{N}\frac{\left|x_{i}-y_{i}\right|}{N\sigma_{i}}$$
  In this case, *x_i_* and *y_i_* represent the *i*th measurement (Table 2, third column) of *N* total measurements for the simulation (*x*) and experiment (*y*; Fig. 3, dashed lines), respectively. Since *d_x,y_* was generated using only one experimental trace for each CIP, the experimental measurement SD *σ_i_* was set to 1 for each measure and, therefore, had no effect on the normalization. The goal here is to use a metric to sort the remaining models relative to the selected experimental traces and so obtain representative models. In this sense, *d_x,y_* serves as a quality metric. The normalized Euclidean distance was evaluated for each CIP, and then the four CIP normalized Euclidean distances were averaged together so that equal importance was given to each signature feature. However, since there are a different number of measurements for each CIP, each measurement did not have equal importance. We felt that this was reasonable to do at this stage, given that it is unknown which specific dynamic regimes are more or less important for IS3 cell function in hippocampal microcircuits.
5. *[Auto] Find representative models:* the models were ranked using the quality metric from smallest (“best”) normalized Euclidean distance to largest. This allowed us to obtain ranked, representative models for each database, albeit qualitative in nature.
6. *[Hand] Adjust parameter space:* the model parameter space was adjusted to reduce the number of models that were eliminated in step 2 of the following iteration (i.e., in cycling back to step 1). This hand-tuning step was performed using clutter-based dimensional reordering (CBDR) plots, a method used previously by Taylor et al. (2006). CBDR plots allow a visualization of the parameter space such that it is easy to see when models in the database are eliminated. Further, these plots visualize the structure of the conductance parameter space by reordering the channel conductance parameters along the *N*-dimensional tensor in order to sort higher-order channels from lower-order channels (i.e., channels that have a greater or smaller impact, respectively, on normalized Euclidean distance). In other words, each pixel in these plots represents a different model with a unique set of VGC conductance values, whose value can be extrapolated from the scale bars on the *x*- and *y*-axes using the maximal conductance values found in Table 6. To minimize space in the CBDR plots where models had been eliminated (Fig. 4*B1–B3*, black pixels) we adjusted the parameter values (in range and resolution) to use in generating the next database (step 1) focusing only on conductance parameter spaces that had generated good models. A cycling process of this is shown in Figure 5. In some instances, we avoided parameter sets for particular reasons (e.g., low-amplitude spikes caused by low transient sodium conductances in SDprox.1; Fig. 5, Cycle 2) even though they were not eliminated in step 2. Once it was no longer possible to increase the parameter space from the inspection of CBDR plots, we moved to step 7.
7. *[Hand] Adjust channel types:* the model channel distributions and/or channel types were adjusted to improve the ability of the models to capture signature experimental features. That is, we analyzed different cases where each case possessed different distributions of channels or had added, altered, or removed channel types, and decided whether there were improvements (Fig. 4*C*). Once manual adjustments were made, we returned to step 1.

**Table 2:** IS3 cell signature features, CIPs, measurements, and model elimination criteria

CIP step	Signature feature	Characteristic measurements of feature	Selected IS3 cell measurement values	Elimination criteria (step 2)
−100 pA	Lack of “sag”	(1) Hyperpolarization *V*_m_ Difference (mean pulse value − mean initial value)	−34.2649 mV	None
		(2) Minimum potential time	58.9000 ms	
		(3) Minimum potential	−112.6709 mV	
		(4) Potential sag	6.7865 mV	
		5) Sag time constant	14.3000 ms	
20 pA	No spiking	(1) Passive depolarization *V*_m_ difference (mean pulse value − mean initial value)	12.4910 mV	If spikes are observed
50 pA	Normal spiking	(1) Active depolarization *V*_m_ difference (mean pulse value − mean initial value)	29.4174 mV	If fewer than three spikes are observed If membrane potential fails to repolarize
		(2) Interspike interval	36.6898 ms	
		(3) First spike time	33.1000 ms	
		(4) Spike voltage threshold mean	−44.3428 mV	
		(5) Spike half-width mean	1.0301 ms	
		(6) Spike amplitude mean	61.7296 mV	
		(7) Spike rate	35.0044 Hz	
		8) Spike maximum afterhyperpolarization mean	6.8820 mV	
		(9) Number of spikes	28.0000	
		(10) Spike frequency adaptation	1.3305	
500 pA	Depolarization block	(1) Depolarization block *V*_m_ difference (final 700 ms mean pulse value − mean initial value)	62.6461 mV	If spikes in the last 700 ms of the CIP are observed If membrane potential fails to repolarize If spikes in the recovery period are observed
		(2) Initial 100 ms spike rate	60.0000 Hz	

For each CIP step (1st column) we note a signature feature that is observed experimentally (2nd column), characteristic measurements that are used to quantify the signature feature (3rd column), characteristic measurement values from the selected IS3 traces (4th column), as well as conditions where models would be rejected for clearly not capturing the experimental feature (5th column). CIP, Current Injection Protocol; *V*_m_, membrane potential.

The overall process is terminated when it is no longer possible improve on the quality metric and the resulting models capture the known experimental data. As these last steps are manual, this termination point is qualitatively determined.

### Uniform channel distributions in soma and proximal dendrites

To incorporate dendritic VGC distributions consistent with the calcium imaging data, we specified channel conductance values that were uniform across the soma and proximal dendrites. We considered this as reasonable since there are no data to support particular distributions (e.g., exponential). A Boltzmann function was used to describe the relationship between the channel conductance and distance from the soma such that non-zero conductances were present only in the soma and proximal dendrites (so, overall, a non-uniform distribution), as follows:

$$f(p)=G-\frac{G}{1+\exp\;(k\times (d-p))}$$

where *f*(*p*) is the distance-dependent conductance, *p* is the distance along the dendrite (in μm), *G* is the desired conductance value in the proximal dendrite (in S/cm^2^), and *k* is a value of 10 (in μm^−1^) in order to allow a sharp decrease in conductance at around *d*, where a *d* of 55, 75, or 95 ensures that the decrease in conductance occurs at around 50, 70 or 90 μm from the soma. Thus, this equation describes the relationship between channel conductance and distance from soma, and the parameters are tuned in such a way that the conductance is uniform at a value of *G* up to a distance of 50, 70, or 90 μm from the soma, after which it drops sharply to 0 S/cm^2^.

### Analysis of the input–output relationship in IS3 cell models

 To understand how the intrinsic properties could affect spike generation in IS3 cells, models were analyzed regarding the required input for spike generation. This was done by defining a synaptic conductance that is activated due to a single presynaptic spike to a target point along the dendritic tree of the model. In this case, a point process is used to describe the synapse as a two-state kinetic scheme to produce synaptic current according to the following:

i=G(v−e)

$$G=weight\;\times \;factor\;\times \;\left(\exp\left(-\frac{t}{tau2}\right)-\exp\left(-\frac{t}{tau1}\right)\right),$$

where *i* is the synaptic current (in nA), *G* is the synaptic conductance (in μS), *v* is the membrane potential (in mV), *e* is the reversal potential (set to 0 mV), weight is synaptic weight (in μS), factor is a NEURON process used to normalize the peak synaptic conductance to 1 (i.e., it ensures that the peak of *G* in response to a single presynaptic spike is equal to weight), *t* is time (ms), *tau1* is the rise time (set to 0.2 ms), and *tau2* is the decay time (set to 2 ms). Although rise time and decay time can vary across different cell types, the values used were chosen based on the measurements in other interneuron types in hippocampus (Tóth, 2010). A full examination of synaptic parameters and their effects on spike generation was beyond the scope of this study.

The synaptic model was incrementally moved to different locations along the dendrite. At each location, the minimum weight value necessary to evoke an action potential at the soma was numerically determined by increasing the weight in increments until the somatic membrane potential surpassed −20 mV in response to a single presynaptic spike. After a somatic spike was recorded, the synapse was then moved to a further dendritic location, and the process was repeated until the entire dendritic arbor was analyzed. Importantly, increasing the weight of a single synapse would eventually lead to a saturation point where (*v – e*) = 0 because *v* would become so depolarized that it was essentially equal to the synaptic reversal potential (*e* = 0 mV) at the dendritic site of the synapse. At this saturation point, no additional current could be generated, regardless of whether weight was further increased (i.e., because *i* = G*(*v* – *e*) = G*0 = 0 pA). To track this, we recorded the change in the membrane potential at the site of the synapse in the first 1 ms following the synaptic event.

## Results

The morphological and membrane properties of IS3 cells have been reported (Chamberland et al., 2010; Tyan et al., 2014); however, the diversity and spatial distribution of VGCs in these cells remain unknown. To simulate how specific types of VGCs located at distinct subcellular domains can affect the IS3 cell input–output properties, we developed a semi-automated strategy (Fig. 2), which generated models mimicking the voltage dynamics seen in IS3 cells. This required the following: (1) experimentally obtained electrophysiological signature features to capture; (2) a compartmentalized model morphology with appropriate passive properties; and (3) initial choices for VGCs.

### Experiment: electrophysiological features of IS3 cells

To use our strategy to estimate IS3 cell VGC composition, we first determined the electrophysiological features that should be captured. For this, we examined voltage traces obtained in response to different current steps, a CIP, in the presence of synaptic blockers to explore IS3 intrinsic cell membrane properties. We note that previous work has shown that CIPs are a simple protocol that sufficiently exposes the dynamics of a cell (Druckmann et al., 2011). We observed the following.

First, during hyperpolarizing steps, IS3 cells exhibited a small membrane potential sag (Fig. 1*B*, red traces). With depolarizing steps, IS3 cells first exhibited a passive response (Fig. 1*B*, green trace) and then began spiking (Fig. 1*B*, blue trace, cell 2). The data obtained here is consistent with a previously recorded rheobase in these cells of 42.8 ± 8 pA (Tyan et al., 2014). Also, these cells exhibited irregular firing patterns, with occasional demonstrations of regular adaptive firing (Fig. 1*B*, blue traces). At higher depolarization steps, progressive spike amplitude adaptation was observed (Fig. 1*B*, blue trace, cell 2), with depolarization-dependent spike block at current steps exceeding 250 pA (Fig. 1*B*, cell 2, orange trace).

With this set of experimental data in mind, we made decisions on which “signature features” to capture in our models. Although there is variability in the electrophysiological data, we needed to make assumptions on how best to constrain the models without being overly restrictive or adding excessive detail that would unnecessarily add computational strain. Thus, we chose the following four signature features that occurred for specific CIP ranges: lack of sag during hyperpolarization; depolarization without spiking; depolarization with normal spiking; and depolarization block (Table 2, second column). Examples of all these signature features are shown in Figure 1*B*. As subthreshold membrane potential fluctuations in the theta range similar to those described in other types of hippocampal interneurons (Chapman and Lacaille, 1999; Morin et al., 2010) were observed in IS3 cells, the VGC types that could be involved were considered when making the initial choices of VGCs. Subthreshold fluctuations and irregular firing were included in the models by the generic addition of noise but were not part of the semi-automated strategy.

For each of the chosen features, we identified characteristic measurements (Table 2, third column). The experimental data are summarized in Figure 3, where each panel is a histogram of a particular measurement from all the experimental traces showing signature features. We selected experimental traces, and the particular CIP values and measurements for them are shown in Table 2 (first and third columns; Fig. 3, histograms, dashed lines). Trace selection was performed manually by going through the experimental data and identifying key electrophysiological regimes. While one might consider automating this aspect, because of biological variability and not knowing the extent of IS3 cell dynamics that might be functionally important, it would be challenging to develop appropriate automated algorithms. Overall, we felt that this was a balanced strategy that would allow us to capture salient IS3 cell features. At the same time, it was necessary to choose experimental traces to be able to automate steps in the process (Fig. 2).

### Experiment: action potential evoked dendritic calcium signals in IS3 cells

Experiments with dendritic patch-clamp recordings and two-photon Ca^2+^ imaging of dendritic Ca^2+^ elevations evoked by backpropagating action potentials (bAPs) have revealed that interneuron types that have a high density of VGCs in their dendrites (in particular, of sodium channels) exhibit non-decrementing bAP-evoked Ca^2+^ signals (Martina et al., 2000; Topolnik et al., 2009; Hu et al., 2010; Camiré and Topolnik, 2014). As direct dendritic recordings from IS3 cells are technically challenging, we performed dendritic two-photon Ca^2+^ imaging of bAP-evoked Ca^2+^ transients (bAP-CaTs) in these cells (Fig. 1*C*–*E*; Table 3). bAP-CaTs were evoked by the somatic injection of three consecutive current pulses (800 pA, 2 ms), and the resulting CaTs were recorded at different points along the dendrite. Our data showed that bAP-CaTs had the same amplitude up until 50 μm from the soma (soma ΔF/F, 0.49 ± 0.08; 50 µm Δ*F*/*F*, 0.53 ± 0.06; *n* = 9, *p* > 0.05, signed rank test) and declined gradually at 150 μm from the soma (Δ*F*/*F*, 0.22 ± 0.08; *n* = 7, *p* < 0.05 compared with somatic CaT, signed rank test). Although the data from individual cells were variable, all cells followed the same trend, exhibiting a significant decline in bAP-CaT amplitude between 50 and 150 μm from the soma (50 μm Δ*F*/*F*, 0.53 ± 0.06; 150 μm Δ*F*/*F*, 0.22 ± 0.08; *n* = 7, *p* < 0.05, signed rank test). These data are consistent with previous observations in different interneuron subtypes (Martina et al., 2000; Golding et al., 2001; Topolnik et al., 2009; Hu et al., 2010; Camiré and Topolnik, 2014) and indicate that specific types of VGCs must be present in IS3 dendrites, and shape the bAP propagation and Ca^2+^ signal generation.

**Table 3:** Statistical tests

Data	Data structure	Type of test	Power
bAP-CaT amplitude at soma vs 50 μm	Unknown	Signed rank test	0.260
bAP-CaT amplitude at soma vs 150 μm	Unknown	Signed rank test	0.028
bAP-CaT amplitude at 50 vs 150 μm	Unknown	Signed rank test	0.043

### Model: starting choices for VGC types and distributions

We considered several different channel types with different distributions. At first, the following four types of channel currents were considered: transient sodium current (*I_Na,t_*), slow delayed rectifier potassium current (*I_Kdrs_*), fast delayed rectifier potassium current (*I_Kdrf_*), and A-type potassium current (*I_Ka_*), since they are known to be present in hippocampal interneurons (Saraga et al., 2003; Lawrence et al., 2006; Hu et al., 2010) and represent a reasonable, minimal set of channel types that should be able to replicate the observed firing patterns in IS3 cells. The channel models used were obtained from a previous OLM cell model developed by Lawrence et al. (2006) without any alterations. The model equations are given in Table 4 along with ModelDB (Hines et al., 2004) and ICGenealogy (http://icg.neurotheory.ox.ac.uk/) reference numbers.

**Table 4:**
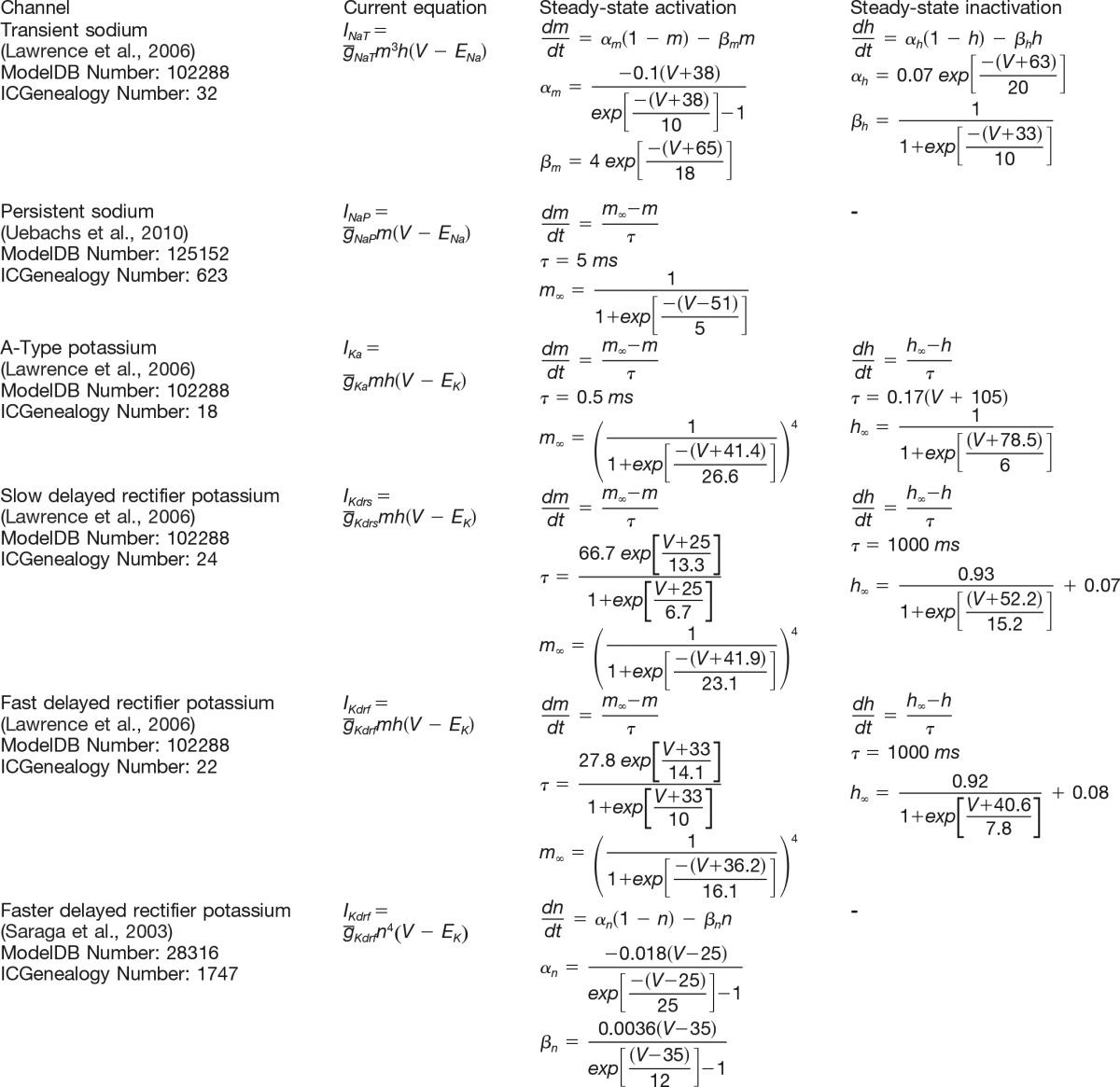
Voltage-gated channel equations

Note that the Na^+^ reversal potential (*E_Na_*) and the K^+^ reversal potential (*E_K_*) are set to values of 50 and −77 mV, respectively, in all simulations. Note also that ModelDB reference numbers and ICGenealogy reference numbers are indicated in the first column. Comparison of channel kinetic traces are available from ICGenealogy.

Intrinsic, subthreshold activities in the theta rhythm and irregular firing were captured in previous interneuron models using *I_Ka_*, persistent sodium current (*I_Na,p_*), and somatically injected white noise current (Morin et al., 2010; Sritharan and Skinner, 2012). Since IS3 cells exhibited similar electrophysiological features (Fig. 1*B*, cell 1, blue trace), somatic *I_Na,p_* was also included. The *I_Na,p_* model was obtained from Uebachs et al. (2010), and the steady-state activation equation parameter values were altered to match the parameters used by Morin et al. (2010) and Sritharan and Skinner (2012). Note that the steady-state mathematical structures for the *I_Na,p_* model are identical in all three of these articles (Morin et al., 2010; Uebachs et al., 2010; Sritharan and Skinner, 2012). Collectively, these channels were the first set of channels used (i.e., S.1; Table 5).

**Table 5:** Summary of channel type combinations and spatial distribution profiles across the morphology of the model

Distribution labels	Soma channel types	Dendrite channel types	Axon channel types
S.1	Persistent sodium Transient sodium A-type potassium Fast delayed rectifier potassium Slow delayed rectifier potassium	None	None
S.2	Persistent sodium Transient sodium A-type potassium Faster delayed rectifier potassium	None	None
SD	Persistent sodium Transient sodium A-type potassium Faster delayed rectifier potassium	Transient sodium A-type potassium Faster delayed rectifier potassium	None

Note that in all cases, each channel has a uniform distribution, whether it is restricted to the soma or distributed across the soma and dendrites.

We now have (1) experimentally obtained electrophysiological signature features to capture, (2) have a compartmentalized model morphology with appropriate passive properties (see Materials and Methods), and (3) made initial choices for VGCs and can proceed with the semi-automated strategy (Fig. 2).

### Model: determining types, densities, and distributions of VGCs using the semi-automated strategy

We investigated a total of 12 different scenarios (not all are shown). This was done in multiples of three: VGCs in the soma only; distributed uniformly in the soma and dendrites; and distributed uniformly in the soma and dendrites with A-type potassium channels restricted to the soma. The first three scenarios contained our initial set of channels, while the second and third triads of scenarios varied the VGC types (see below). The fourth triad of scenarios possessed L-type and T-type calcium channels (see Discussion). As most of these scenarios were not able to encompass the determined electrophysiological features, they are not described any further.

We describe and present three of these scenarios in detail (Table 5), as follows: the initial set of channels in the soma only (S.1); the set of channels with faster potassium kinetics in the soma only (S.2); and the set of channels with faster potassium kinetics in the soma and dendrites (SD). Focusing our results on these three scenarios allows us to show the clear progression from our initial assumptions to our improved models, and also allows us to demonstrate the impact of dendritic channels. Note that distribution labels containing an “S” means somatic channels, “D” means dendritic channels, and “*.x*” denotes different versions of similar distributions (e.g., different channel types). We also investigated channels in specific dendritic subregions. These models possessed the label “SDprox”, meaning that they had channels in the soma and proximal dendrites.

For S.1 (Fig. 4 *A1*,*B1*), in visualizing the parameter space using the CBDR plots, we found that a fairly large parameter space yields models that are capable of eliciting the key features of IS3 cells (i.e., colored pixels represent models that were not eliminated in step 2). However, looking more closely at the histograms of measurements from the database of model populations, we noticed that they could fall completely outside those of the experimental histogram measurements. For example, the spike threshold was too low, the half-width was too large (Fig. 4*C*, left histogram), and the spike afterhyperpolarization was too large. In addition to this, the depolarization height in the membrane potential during depolarizing current injections was also too small (i.e., not just in the +50 pA step but also in the +20 pA and +500 pA steps). To quantify this “depolarization height” feature, we included a measure that calculates the difference between the average membrane potential before the current injection and the average membrane potential during the current injection (Table 2, third column).

To illustrate the advantage of this semi-automated strategy over a purely hand-tuned strategy, we show the conductance ranges at the start and end (steps 0 and 7, respectively) of the cyclic approach for the different conductances shown in Table 6. Note that they vary in their range, and in their maximum and minimum values. Determining this by hand tuning only would be tedious, time consuming, and potentially without success, as changing any particular conductance and still capturing salient features may require shifting other conductances into other ranges.

**Table 6:** Summary of the starting and final conductance ranges found using the semi-automated strategy for S.1, S.2, and SD

Distribution label	*G_Na,t_* (S/cm^2^)	*G_Na,p_* (S/cm^2^)	*G_Ka_* (S/cm^2^)	*G_Kdrf_* (S/cm^2^)	*G_Kdrs_* (S/cm^2^)
S.1 start	0.08, 0.12, 0.16, 0.20	0.0002, 0.0003, 0.0004	0.05, 0.10, 0.15	0.04, 0.08, 0.12	0.04, 0.08, 0.12
S.1 final	0.16, 0.18, 0.20, 0.22	0.0001, 0.0002, 0.0003	0.05, 0.10, 0.15	0.03, 0.07, 0.11	0.03, 0.06, 0.09
S.2 start	0.2, 0.225, 0.25	0.0001, 0.00015, 0.0002	0.15, 0.20, 0.25	0.8, 0.9, 1.0(faster)	0
S.2 final	0.2, 0.225, 0.25	0.00005, 0.00010, 0.00015	0.15, 0.20, 0.25	0.95, 1.0, 1.05(faster)	0
SD start	0.04, 0.05, 0.06	0.0002, 0.0004, 0.0006	0.06, 0.08, 0.1	0.05, 0.10, 0.15(faster)	0
SD final	0.04, 0.05, 0.06	0.0002, 0.0004, 0.0004	0.06, 0.08, 0.1	0.1, 0.13, 0.16(faster)	0
SDprox.1 start	0.05, 0.075, 0.1(50, 70, 90 μm)	0.00005, 0.00010, 0.00015	0.03, 0.05, 0.07(50, 70, 90 μm)	0.2, 0.3, 0.4(faster)(50, 70, 90 μm)	0
SDprox.1 final	0.07, 0.0725, 0.075(50, 70, 90 μm)	0.00005, 0.000075, 0.0001	0.03, 0.05, 0.07(50, 70, 90 μm)	0.25, 0.275, 0.3(faster)(50, 70, 90 μm)	0
SDprox.2 start	0.055, 0.1025, 0.15(50, 70, 90 μm)	0.00005, 0.00010, 0.00015	0.03, 0.05, 0.07	0.2, 0.3, 0.4(faster)(50, 70, 90 μm)	0
SDprox.2 final	0.055, 0.060, 0.065(50, 70, 90 μm)	0.00005, 0.00010, 0.00015	0.03, 0.05, 0.07	0.27, 0.295, 0.32(faster)(50, 70, 90 μm)	0

Note that for the SDprox.1 and SDprox.2 distributions, we also investigated channels that were uniform in soma and dendrites up until 50, 70, and 90 μm from the soma. The choice of the number of conductance values to use was determined by balancing parameter space resolution against computational speed.

In the absence of any particular knowledge of VGCs in IS3 cells or of particular IS3 cell dynamics of functional importance, we constrained our models with a few chosen signature features and a quality metric (see Materials and Methods). This meant that a number of different current injection steps from experiment were lumped together for a given feature (e.g., for the depolarization block feature, current steps of 450–700 pA were used, giving ranging histograms; Fig. 3*Q*,*R*). In essence, we used the experimental histogram measurements (Fig. 3) as a guide. We felt that it made more sense to simply compare each measurement histogram independently when making choices on channel types, distributions, and conductance ranges, as our goal is to obtain starting reference models that would suggest the types, densities, and distributions of VGCs. In this sense, we considered biological variability in a manual fashion (i.e., steps 6 and 7).

#### Subsequent investigations (step 7)

In noting that particular measurements could not be captured with the initial choice of VGCs, we considered changes in the set of VGCs being used (Fig. 4*C*, flowchart). In particular, we had observed through hand tuning that by using delayed rectifier potassium channels with faster kinetics, the spike half-width and afterhyperpolarization were decreased, leading to an improvement in the ability of the models to resemble the experimental signature features. This, in turn, allowed for VGC conductance densities that increase the depolarization height in the membrane potential during depolarizing currents. In terms of using faster delayed rectifier potassium kinetics, one option is to remove the slow delayed rectifier channels from the model, and the other is to use faster time constants in the fast delayed rectifier channel model.

With this in mind, we applied the semi-automated strategy, but without a slow delayed rectifier potassium channel and with a more generic fast delayed rectifier channel containing faster delayed rectifier potassium channel kinetics (channel model was taken from Saraga et al., 2003). The equations are given in Table 4. We show the scenario with only somatic channels (i.e., S.2) and with channels uniformly distributed across the soma and dendrites (i.e., SD; Table 5; Fig. 4*A2*,*A3*,*B2*,*B3*). For both channel distributions, measurement distributions generated from the models in each database showed more overlap with the experimental measurement distributions when compared with S.1 (Fig. 4*C*). For these channel distributions, we obtained an increase in the depolarization height of the membrane potential during depolarizing currents. However, it was not as large as that seen in the experimental recordings. Specifically, during the +50 pA step in the experimental trace, we see a depolarization height of 29.42 mV (Fig. 1*B*, cell 2) and an experimental trace distribution ranging from 15 to 34 mV (Fig. 3*G*). While the S.1 top model fell outside this range, with a depolarization height of 10.83 mV (Fig. 4*A1*), both S.2 and SD top models fell closer to the experimental range with depolarization heights of 21.37 and 14.55 mV, respectively (Fig. 4*A2*,*A3*). Other measurements such as the spike half-widths and the spike afterhyperpolarizations were also improved. Thus, we were able to distinguish that the changes made in S.2 and SD from S.1 led to measured improvements in the models.

In terms of distinguishing between S.2 and SD, we looked at several additional factors. For example, we looked at the first spike time distributions in each database (Fig. 4*C*, right). Experimental recordings indicate that during regular spiking regimes, the first spike time of the current injection is usually early on (i.e., between 0 and 40 ms). Although S.2 ranges from spikes starting as early as 40 ms to ∼120 ms, SD mainly ranges from 50 to 350 ms. This indicates that spike onset is often too late in all the models, but that S.2 performs better. Another factor that distinguished S.2 from SD is the presence of spike amplitude decreases during the regular spiking regime (i.e., at +50 pA). Whereas the models generated in SD have spike amplitudes that increase during regular spiking, S.2 has the opposite: spike amplitudes that start off large and then decrease over the course of the current injection. This is observed in the top model for S.2 in Figure 4*A2*, compared with the experimental recording (Fig. 1*B*, cell 2, blue trace). As mentioned previously, decreasing amplitudes is a common feature observed in the experimental data, and it is therefore an important feature to replicate in our models.

The determined conductance value ranges are listed in Table 6 (see final ranges). We note the following: in SD, distributing the channels uniformly across the dendrites requires an overall decrease in conductance values (i.e., with the exception of persistent sodium, which is already quite small). For the most part, the conductance value ranges are similar among S.1, S.2, and SD, with the exception of the potassium channels, which generally have higher conductance values in S.2 and SD, likely due to the removal of the slow delayed rectifier channel.

#### Delayed rectifier potassium channel subunit analysis

To investigate the predictions generated by the models, we sought to examine the delayed rectifier potassium channel subunit composition in IS3 cells. Specifically, we wanted to know whether fast or slow delayed rectifier potassium channels existed in IS3 cells.

For slow delayed rectifier potassium channel subunit composition, it is generally known that Kv2.1 or Kv2.2 combine with Kv5.1, Kv6.4, or Kv9.1 to Kv9.3 (Lien et al., 2002; Coetzee et al., 1999). Fast delayed rectifier potassium channels, on the other hand, are generally known to be composed of Kv3.1 and/or Kv3.2 subunits, of which Kv3.1 composition yields faster time constants (Hernández-Pineda et al., 1999; Lien et al., 2002). To get an idea of expression of these subunits in the stratum radiatum (SR) of the hippocampus (i.e., the layer where IS3 cell bodies and the large majority of its dendrites are located), we examined the *in situ* hybridization data from mouse brain slices using the Allen Mouse Brain Atlas (Lein et al., 2007; for full documentation, see http://help.brain-map.org/display/mousebrain) as a point of reference. For the slow delayed rectifier subunits, it appeared as though there is very little expression, if any, in the SR or stratum lacunosum-moleculare (SLM). For fast delayed rectifier subunits, it appeared as though Kv3.1 subunit expression was much more prominent in the SR than Kv3.2 subunits.

**Figure 6.**
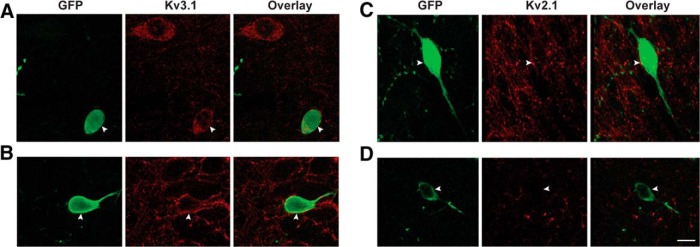
Kv3.1 and Kv2.1 expression in putative IS3 cells. ***A***, ***B***, Immunohistochemistry data showing GFP (green; left) and Kv3.1 expression (red; middle) in the stratum radiatum (***A***) and pyramidale (***B***) of a VIP-GFP mouse. Note the presence of Kv3.1 membrane labeling in the soma and proximal dendrites of VIP- expressing cells. ***C***, ***D***, Immunohistochemistry data showing GFP (green; left) and Kv2.1 expression (red; middle) in the stratum pyramidale (***C***) and radiatum (***D***) of a VIP-GFP mouse. Scale bar, 10 μm.

Using immunohistochemical analysis of the subunit localization in soma and dendrites of IS3 cells, we were able to examine whether Kv2.1 and Kv3.1 were expressed. We found that Kv3.1 is present in both the soma and proximal dendrites of putative IS3 cells in stratum pyramidale (SP) and SR (Fig. 6*A*,*B*). However, we found no evidence for Kv2.1 expression in IS3 cells residing in SR (Fig. 6*D*), and its sparse expression in IS3 cell bodies located within SP that, based on the punctate pattern around the cell body (Fig. 6*C*), could be attributed to axonal boutons contacting IS3 cells (Fig. 6*C*,*D*). These data indicate that Kv3.1, but not Kv2.1, is expressed in IS3 cells. Thus, the model predictions that delayed rectifier potassium channels with faster kinetics are present and slow delayed rectifier potassium currents are absent in IS3 cells were confirmed.

### Model adjustments to consider spike propagation

**Figure 7.**
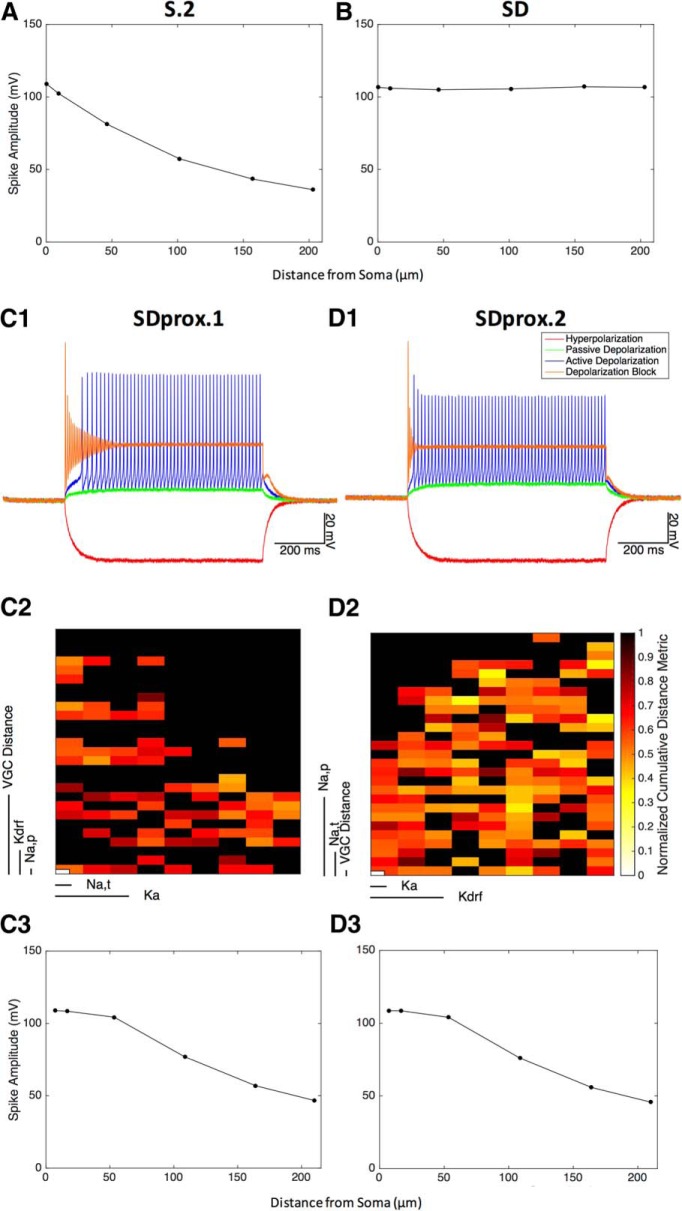
VGC distributions in proximal dendrites. ***A***, Action potential amplitude deterioration along a dendrite section (tree 1) in the S.2 top model following a somatic 800 pA current injection for 2 ms (*G_Na,t_* soma = 0.25 S/cm^2^; *G_Na,p_* soma = 0.0001 S/cm^2^; *G_Ka_* soma = 0.15 S/cm^2^; *G_Kdrf_* soma = 1 S/cm^2^). ***B***, Action potential amplitude deterioration along a dendrite section (tree 1) in the SD top model following a somatic 800 pA current injection for 2 ms (*G_Na,t_* soma/dendrites = 0.06 S/cm^2^; *G_Na,p_* soma = 0.0002 S/cm^2^; *G_Ka_* soma/dendrites = 0.1 S/cm^2^; *G_Kdrf_* soma/dendrites = 0.1 S/cm^2^). ***C1***, SDprox.1 top model with channels that are uniform from the soma until the first 70 μm of the dendrites using the Boltzmann function. Conductance values are as follows: *G_Na,t_* soma/dendrites = 0.07 S/cm^2^; *G_Na,p_* soma = 0.000075 S/cm^2^; *G_Kdrf_* soma/dendrites = 0.25 S/cm^2^; *G_Ka_* soma/dendrites = 0.07 S/cm^2^. Note that the current injection protocol is −100, +20, + 50, and +500 pA. ***C2***, Parameter spaces for SDprox.1, as visualized using CBDR. Note that 70 of 243 models did not get rejected. ***C3***, Action potential amplitude deterioration along a dendrite section (tree 1) in the above SDprox.1 top model following a somatic 800 pA current injection for 2 ms. ***D1***, SDprox.2 top model with channels that are uniform from the soma until the first 70 μm of the dendrites using the Boltzmann function. Conductance values are as follows: *G_Na,t_* soma/dendrites = 0.055 S/cm^2^; *G_Na,p_* soma = 0.00015 S/cm^2^; *G_Kdrf_* soma/dendrites = 0.295 S/cm^2^; *G_Ka_* soma = 0.07 S/cm^2^. Note that the current injection protocol is −100, +20, + 50, and +500 pA. ***D2***, Parameter spaces for SDprox.2, as visualized using CBDR. Note that 146 of 243 models did not get rejected. ***D3***, Action potential amplitude deterioration along a dendrite section in the above SDprox.2 top model following a somatic 800 pA current injection for 2 ms.

So far, it seems as though S.2 models present a reasonable scenario for IS3 cell channel types and distributions. On the other hand, if we consider S.2 as the most reasonable models, this would indicate that channels in IS3 cells are restricted to the soma and that dendritic channel distributions are not a suitable aspect to capture normal IS3 cell electrophysiological activity. However, given that our immunohistochemical analyses indicate that potassium channels are present in the dendrites, this is not appropriate. Further, our models so far only considered uniform channel distributions in the whole dendritic tree. Thus, we turn to our Ca^2+^ imaging data and consider the spatial profile of the spike amplitudes, as derived from the bAP-CaTs profile, in two of our models: the highest-ranking model from S.2 and the highest-ranking model from SD. In particular, we used a methodology similar to that used experimentally by injecting somatic current (800 pA, 2 ms) to induce bAPs. We then qualitatively compared the bAP amplitude decay with distance from soma to the calcium signal amplitude decay seen in experimental recordings. As can be observed in Figure 7*A*, although S.2 bAP amplitudes decay, bAPs are still seen at large distances from the soma (i.e., small ∼40 mV amplitude spikes are still observed at 200 μm from the soma).

At the 150 μm location, these amplitudes are 40% of the amplitude at soma, which is comparable to the average calcium signal decay of 50% at 150 μm (Fig. 1*E*). However, there is a lack of calcium signal decay in the first 50 μm of dendrite, which is not comparable to the S.2 models. SD on the other hand does not show any spike amplitude decay (Fig. 7*B*), regardless of the distance from soma, which is expected because of the high density of VGCs distributed uniformly across the dendrites. Altogether, these results suggest the presence of VGCs in at least the first 50 μm of dendrites.

#### Channels only in soma and proximal dendrites: SDprox.1 and SDprox.2

To better capture the experimental data of calcium signal decay, we adjusted our models to have dendritic channels only in the proximal dendrites (i.e., up to 50–100 μm from the soma; see Materials and Methods) and used the semi-automated strategy to estimate the required VGC conductance densities to be able to capture the electrophysiological signature features. In addition to looking at variations in maximal conductance values, we considered channels at different distances from soma (i.e., 50, 70, or 90 μm; Fig. 7*C2*,*D2*). We also considered model variants where A-type potassium channels were confined to the soma (SDprox.2) or uniform in the soma and proximal dendrites (SDprox.1). Models in the databases for SDprox.1 (Fig. 7*C2*) and SDprox.2 (Fig. 7*D2*) can have channels at 50, 70, or 90 μm (Fig. 7, denoted as “VGC Distance” in the CBDR plots) on top of having different VGC conductance densities.

In Figure 7, *C1* and *D1*, we see the simulated voltage traces from top models from the SDprox.1 database and the SDprox.2 database. See Table 6 for conductance ranges, and Table 7 for top model conductance values and VGC distances along dendrites. From the traces for both of these models, we see that they appear to be very similar in quality to the S.2 models, which shows that a scenario with VGCs in the proximal dendrites (preferably at 50 and 70 μm) is capable of capturing the electrophysiological features. Notably, from the measurement histograms we see that these databases, similar to the S.2 database, fall within many of the experimental measurement distributions. Also, using the same recording procedure (i.e., an 800 pA, 2 ms duration somatic current injection; Fig. 7*C3*,*D3*), we found that bAP amplitude decay is attenuated in the first 50 μm of dendrites in these two scenarios, unlike the continuous spike amplitude decay seen in the S.2 model. With these developed models in hand, we next determined the location and amount of synaptic input necessary for spiking to occur.

**Table 7:** Summary of starting hand-tuned conductance values and top model conductance values from SDprox.1 and SDprox.2

Model	Distribution	*G_Na,t_* (S/cm^2^)	*G_Na,p_* (S/cm^2^)	*G_Ka_* (S/cm^2^)	*G_Kdrf_* (faster kinetics)(S/cm^2^)
SDprox.1 hand tuned	Uniform across soma and first 50 μm of dendrites(*G_Na,p_*: soma only)	0.075	0.0001	0.05	0.3
SDprox.1 top model	Uniform across soma and first 70 μm of dendrites(*G_Na,p_*: soma only)	0.07	0.000075	0.07	0.25
SDprox.1 second best model	Uniform across soma and first 50 μm of dendrites(*G_Na,p_*: soma only)	0.075	0.000075	0.07	0.3
SDprox.1 third best model	Uniform across soma and first 70 μm of dendrites(*G_Na,p_*: soma only)	0.07	0.00005	0.07	0.25
SDprox.2 hand tuned	Uniform across soma and first 50 μm of dendrites(*G_Na,p_* and *G_Ka_*: soma only)	0.07	0.0001	0.05	0.3
SDprox.2 top model	Uniform across soma and first 70 μm of dendrites(*G_Na,p_* and *G_Ka_*: soma only)	0.055	0.00015	0.03	0.295
SDprox.2 second best model	Uniform across soma and first 70 μm of dendrites(*G_Na,p_* and *G_Ka_*: soma only)	0.06	0.00015	0.05	0.295
SDprox.2 third best model	Uniform across soma and first 90 μm of dendrites(*G_Na,p_* and *G_Ka_*: soma only)	0.06	0.00015	0.07	0.32

### Exploration of required input for spike generation in IS3 cell models

It was previously shown that unitary synaptic conductance at IS3–OLM cell synapses is small, but at the same time, the synchronous recruitment of many IS3 cells through optogenetic stimulation could control the OLM cell firing at theta frequencies (Tyan et al., 2014). To understand how incoming inputs can integrate to trigger spiking in IS3 cells, we used our developed multi-compartment models of IS3 cells. As described above, these were S.2 models with VGCs only in the soma, SD models with VGCs uniformly distributed in the dendrites, and SDprox.1 and SDprox.2 models that had channels in the proximal dendrites. Only the SDprox.1 and SDprox.2 models were appropriate for capturing the salient features of IS3 cells and spike attenuation along the dendrites as well as the immunohistochemical analyses, indicating the presence of fast delayed rectifier potassium channels in the proximal dendrites. To explore the impact of different spatial locations on the input attenuation along the dendritic tree of our models, we first performed an electrotonic analysis.

#### Electrotonic analysis of passive model

**Figure 8.**
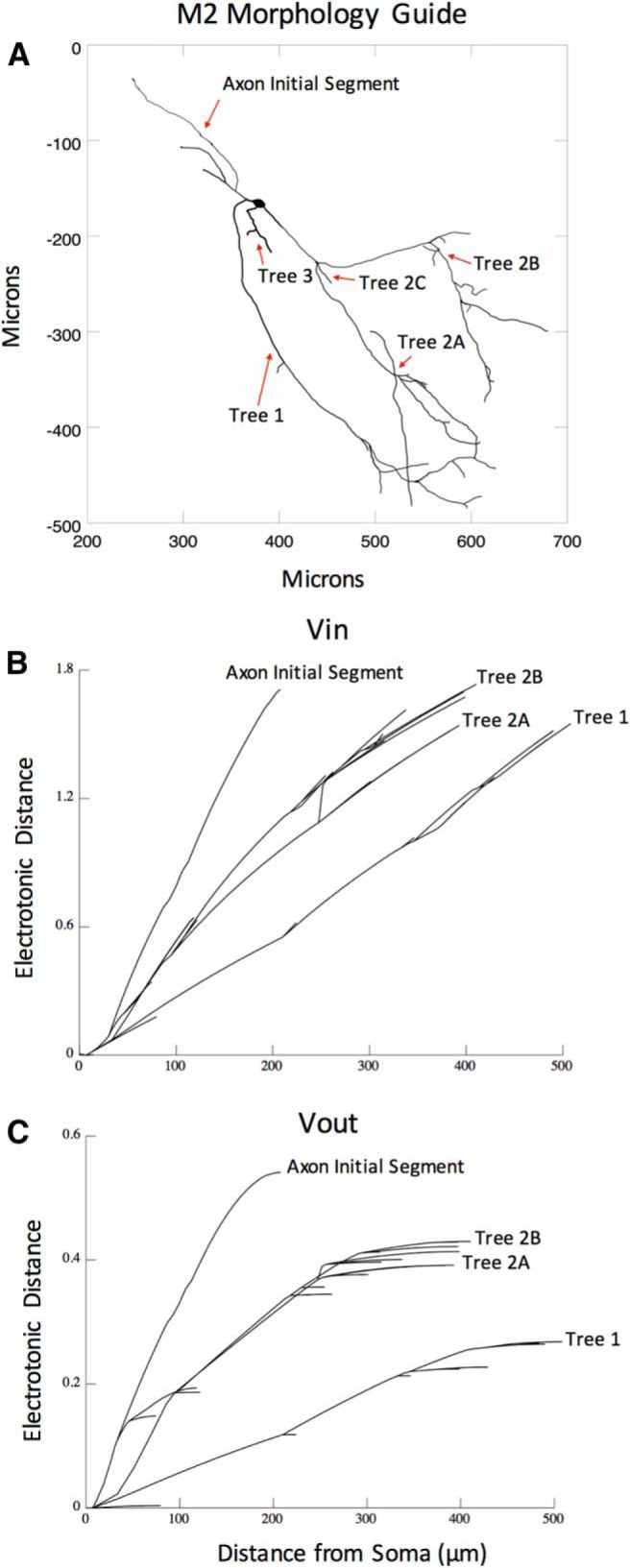
Electrotonic analysis of the M2 morphology. ***A***, A guide of the M2 morphology designating all of the morphological subsections, as well as showing both the spatial scale and diameter. ***B***, Electrotonic distance along the dendritic arbor for voltage flowing into the soma. ***C***, Electrotonic distance along the dendritic arbor for voltage flowing away from the soma. Note that the electrotonic distance is equal to the log value of attenuation, where attenuation is measured as voltage upstream/voltage downstream. More specifically, voltage upstream is an applied 1 mV signal, and voltage downstream is the downstream response to the 1 mV signal. In this sense, electrotonic distances >1 would imply a 10-fold attenuation in the signal.

We used the Electrotonic Analysis tools in NEURON to compute the electrotonic distance throughout the entire dendritic arbor of the passive model (see Materials and Methods). Figure 8*A* shows the morphology with labeled trees, while the number of branch points, surface area, most distal length, and summed length from all branches are given in Table 8. Tree 1, for example, has less branching and longer sections, and has a larger surface area than the other dendritic trees, 2A and 2B.

**Table 8.** Morphological analysis of the IS3 cell multi-compartment model

Tree	Number of branching points	Surface area* (μm^2^)	Maximum distal length (μm)	Summed length from all branches (μm)
1	7	2463.79	508.41	749.40
2A	8	2385.98	399.00	789.62
2B	9	1982.60	410.84	704.49
2C	1	372.21	121.80	114.51
3	1	447.42	79.68	85.28
Axon	2	859.35	208.04	317.45

*Note that to compute the surface area, the effective area was computed for each compartment via a trapezoidal integration across the compartment length using the NEURON area(x) function.

In the axon initial segment and trees 1, 2A, and 2B, we found that electrotonic distance increases with distance from soma (Fig. 8*B*,*C*), which indicates signal attenuation throughout the model. We also observed differences between trees in the magnitude of electrotonic distance. For example, an input into the dendrite of tree 1 versus tree 2A at 300 μm is attenuated by an electrotonic distance of 0.9 versus 1.3 (Fig. 8*B*). Similarly, a somatic input attenuates by an electrotonic distance of 0.2 versus 0.4 when measured at 300 μm along tree 1 versus tree 2A (Fig. 8*C*). 

This electrotonic distance appears to be smaller in dendritic trees with less branching and larger surface areas (Table 8, tree 1). For example, while tree 2A showed more attenuation (i.e., larger electrotonic distances) than tree 1, we also noted that tree 1 had less branching (seven branching points) and a larger surface area (2463.79 μm^2^; determined by section diameters and lengths) than tree 2A (eight branching points and 2385.98 μm^2^; Table 8). For similar reasons, both tree 1 and tree 2A showed less attenuation than tree 2B (nine branching points and 1982.60 μm^2^; Fig. 8, Table 8). Although a greater level of attenuation was observed in the remaining axon segment, this is likely because of the adjusted passive parameters that were used to compensate for the removal of the axon. Collectively, these results suggest that synapses located on dendritic trees with higher branching and smaller surface areas (e.g., trees 2A and 2B relative to tree 1) are less likely to elicit spikes at large distances from the soma because of a greater level of attenuation (Fig. 8).

#### Synaptic inputs

**Figure 9.**
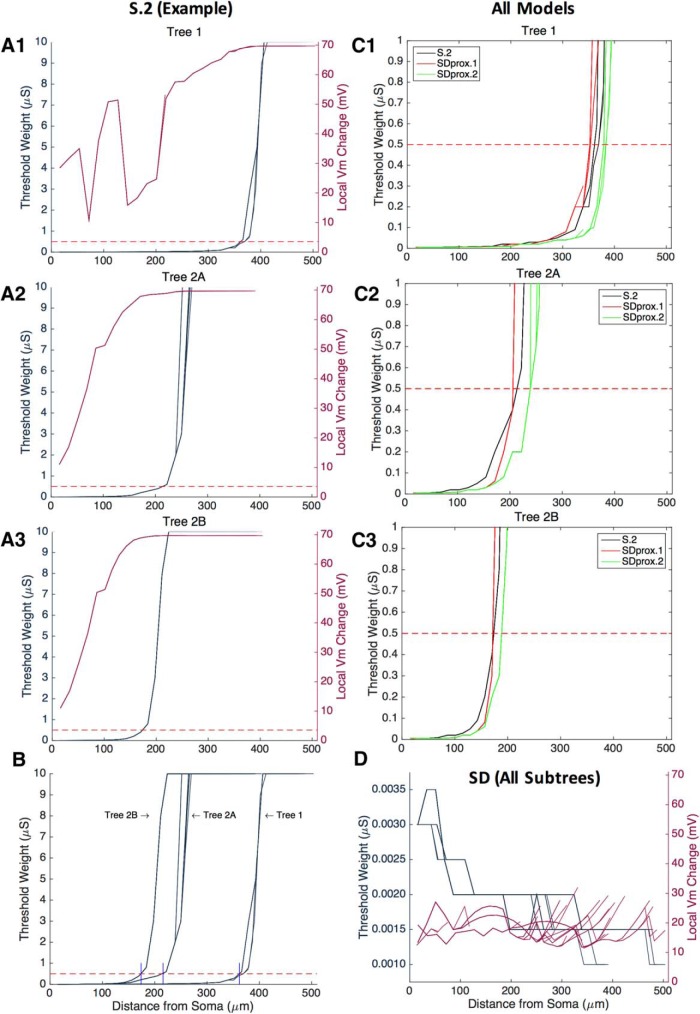
Threshold weight with distance from soma in top models. ***A1–A3***, Left *y*-axes, Synaptic weight threshold (in μS) necessary to evoke a somatic spike in response to a single presynaptic spike applied incrementally at different points along the dendritic arbor for the S.2 top model. Right *y*-axes, S.2 top model local changes in membrane potential (i.e., maximum potential − minimum potential in the first 1 ms following the presynaptic spike) at the site of the synapse, where synaptic current saturation is reached once the local membrane potential reaches the reversal potential (i.e., an increase of ∼70 mV). Note that tree 1 is plotted in ***A1***, tree 2A is plotted in ***A2***, and tree 2B is plotted in ***A3***. The plots show that the location along the dendrites where there is an exponential increase in threshold weight (i.e., dashed red line at around 0.5 μS) for each main dendritic tree approximately co-locates with the location along the dendrites where synaptic current saturation occurs. ***B***, S.2 top model synaptic threshold weights (in μS) in all dendritic trees. Plot shows that there are differences in the location along the dendrites where there is an exponential increase (i.e., dashed red line) in the threshold weight, depending on the dendritic tree of interest. ***C1–C3***, Relative to passive dendrites (S.2 top model), active dendrites with A-type potassium channels in the first 70 μm of dendrites (SDprox.1 top model) show a decrease in the distance from the soma at which the exponential increase in threshold weight is observed. On the other hand, active dendrites in the first 70 μm of dendrites with A-type potassium channels restricted to the soma (SDprox.2 top model) shows an increase in the distance from the soma at which the exponential increase in threshold weight is observed. This is shown in all main trees (***C1–C3***). ***D***, Note that the SD top model does not reach input saturation, and the amount of input to elicit a somatic spike does not increase exponentially in any of the dendritic trees.

We simulated single presynaptic spikes that give rise to synaptic inputs at different locations along the dendritic arbors (see Materials and Methods) of the S.2 top model, the SD top model, the SDprox.1 top model, and the SDprox.2 top model. These simulations allowed predictions of the minimum synaptic weights (threshold weight) necessary to evoke spikes at the somata of IS3 cells (Fig. 9).

For the S.2, SDprox.1, and SDprox.2 models, there were distinct dendritic points where threshold weight rose sharply (Fig. 9; i.e., the point marked by the bottom red dashed line at 0.5 μS). These points correspond to the level of synaptic current at which the membrane potential approached the reversal potential (i.e., 0 mV). In other words, they correspond to when the increase in membrane potential following an excitatory synaptic event was ∼70 mV (Fig. 9*A1–A3*). We identified this “saturation” by plotting the maximal change in membrane potential (i.e., maximum potential − minimum potential) at the synapse location within the first 1 ms of the presynaptic spike (Fig. 9*A1–A3*, S.2 top model). When the change in membrane potential had a magnitude equal to the absolute value of the resting membrane potential (∼70 mV), the synapse was not generating any additional current, regardless of the magnitude of the weight. This occurred at synaptic weights beyond ∼0.5 μS, as indicated by the red dashed line.

Furthermore, we found that for each main dendritic tree (i.e., trees 1, 2A, and 2B), the point at which the threshold weight increased sharply (red dashed line) occurs at a different distance from the soma, as shown in the plot of Figure 9*B* for the S.2 model. In these plots, each vertical blue line marks a main dendritic tree (Fig. 8*A*, subset morphologies; Table 8, morphological analysis). Synaptic inputs to tree 1, with its smaller number of branching points and longer sections than other trees, generated somatic spikes up until 350–400 μm. Synaptic inputs to tree 2A could elicit somatic spikes up until 200–250 μm. Tree 2B had the largest amount of branching and the smallest surface area, and elicited spikes up until 150–200 μm. These observations make sense following our electrotonic analyses in which trees 2A and 2B had more attenuation relative to tree 1.

In Figure 9*C1–C3*, we show differences among S.2, SDprox.1, and SDprox.2, noting that SDprox.1 and SDprox.2 do not have passive dendrites like S.2. In SDprox.1, some of the dendritic trees show a slight decrease in the distance from soma at which the threshold weight increases past 0.5 μS, suggesting that the dendritic channel distribution and densities (i.e., *I_Na,t_*, *I_Kdrf_*, and *I_Ka_*) do not appreciably decrease the required input for spike generation when compared with a passive dendritic scenario (i.e., S.2). However, in SDprox.2 (Fig. 9*C1–C3*), the location at which synaptic input could generate spikes was shifted forward by ∼10 μm in all of the branches, indicating that, by not including A-type potassium channels in the dendrites, spike generation is facilitated.

Finally, in the SD top model, we found that the required input (threshold weight) for spike generation was minimal (i.e., ≪0.5 μS) throughout the dendritic arbor and even decreased with distance from the soma (Fig. 9*D*, left *y*-axis). This is likely because synaptic saturation is never reached in the SD model (Fig. 9*D*, right *y*-axis). This suggests that uniform VGCs across the dendritic arbor could amplify distal inputs over proximal ones. However, since the threshold weight is fairly small across synaptic locations, it seems unlikely that any significant preference for distal inputs over proximal inputs would be observed.

## Discussion

We have presented an efficient, semi-automated strategy to estimate the active VGC properties of identified single neurons that uses electrophysiological data and dendritic calcium imaging to constrain various possibilities. Using this approach, we generated databases of models that mimic the various features seen in IS3 cell experimental recordings. From these models, we obtained estimates of the VGC types, densities, and distributions that may be found in IS3 cells. Specifically, our models predict that fast potassium channel kinetics play a large role in generating appropriate electrophysiological activity, and their presence was confirmed with immunohistochemical analyses. Our models also predict that VGCs in the proximal dendrites best facilitate spike generation when A-type potassium channels are restricted to the soma. We note that, although the approach still involves a fair amount of hand tuning, the automated cycling brings forth a structured aspect that ensures that the included VGCs have conductance values that are balanced relative to each other so as to produce the observed electrophysiological features. In summary, this approach is a much more efficient approach (relative to hand tuning) that takes advantage of automated model databases and visualization tools, successfully predicting the presence of particular VGCs in IS3 cells.

Notably, in all cases where starting values were recorded, the starting hand-tuned parameters and ranges always differed from the final top model conductance values and ranges (Tables 6, 7). The particular VGC conductance differences could be small or large, but the improvement in model quality between parameter refinement cycles is apparent when looking at the CBDR plots between cycles (Fig. 5). Specifically, these plots highlight how the different channel-type choices and conductance values have clear impacts on model database quality. As such, the predicted relative conductance values are meaningful and are not simply a particular hand-tuned choice.

### Proximal dendritic distributions of VGCs on IS3 cells

When analyzing the minimal synaptic weights necessary to elicit a spike along the dendritic arbor of our models, we found that these weights are minimized when the models possess uniform distributions of channels along the dendrites. Interestingly, for the models with VGCs in the proximal dendrites, the synaptic weights necessary to elicit a somatic spike are minimized when A-type potassium channels are restricted to the soma. This makes sense since the presence of additional outward potassium currents in the dendrite would effectively require larger, excitatory inputs to generate a spike. Furthermore, previous experimental (Magee et al., 1998; Magee, 2000; Cai et al., 2004; Yang et al., 2015) and modeling (Tigerholm et al., 2013) work shows that A-type potassium channels contribute to sublinear summation of dendritic spikes, thus dampening dendritic excitability during dendritic integration in pyramidal cells.

In terms of how well these different model types replicate IS3 cell features, we found that uniform distributions of channels along the dendrites (i.e., SD models) do not acceptably reproduce the electrophysiological features of IS3 cells, whereas passive dendritic cases (i.e., S.2 models) do. However, S.2 models have inappropriate spike amplitudes and propagation when considering dendritic Ca^2+^ imaging data from IS3 cells. For these reasons, we suggest that the models with VGCs in proximal dendrites (i.e., SDprox) offer the most likely scenario for IS3 cell active properties, particularly when VGCs extend up to 70 μm from the soma, since this is seen in the top models from the SDprox cases. This type of dendritic ion channel distribution would fall somewhere between the ion channel distributions found in basket cells and OLM cells. Particularly, in previous basket cell modeling work, low bAP amplitudes in dendrites have been associated with a high potassium-to-sodium channel ratio (Hu et al., 2010). Conversely, in previous OLM cell models, the ratio of dendritic sodium channels to potassium channels was not low, leading to high bAP amplitudes across most of the dendrites (Martina et al., 2000; Saraga et al., 2003). Similar results for both basket and OLM cells were obtained in previous dendritic Ca^2+^ imaging studies investigating bAP-CaTs amplitudes (Topolnik et al., 2009; Camiré and Topolnik, 2014). Since the bAP-CaTs observed for IS3 cells (Fig. 1*E*) fall somewhere in between what is seen in basket and OLM cells, it might follow that IS3 dendritic ion channel densities fall somewhere in between as well, as is indicated by our models.

Additionally, it is known that in pyramidal cells, A-type potassium channel conductance values are much larger in distal apical dendrites than in proximal dendrites and soma (Magee et al., 1998; Golding et al., 2001). Although we did not investigate fully nonuniform dendritic ion channel distributions in our models, we explored two possible scenarios for A-type potassium channels: A-type potassium channel in soma and proximal dendrites (SDprox.1) versus that restricted to soma (SDprox.2). In these models, we explain a reduction in bAP amplitude by only having ion channels uniformly spread across a portion of the dendrites. In reality, this could be due to an increase in A-type potassium with distance from soma similar to what is seen in pyramidal cells (Golding et al., 2001). On the other hand, it could be similar to basket cells in that, past a certain distance from soma, the ratio of potassium to sodium channels becomes larger, resulting in a decay of bAP amplitude (Hu et al., 2010). Of course, the question of whether or not A-type potassium channels are present in IS3 dendrites remains to be determined.

Proximal dendritic VGC distributions on IS3 cells could serve several functions. For example, having large densities of VGCs in the proximal dendrites of IS3 cells may facilitate CA3 input gating, spike backpropagation, and induction of Hebbian forms of long-term potentiation at SC synapses (Golding et al., 2002; Topolnik, 2012). Accordingly, IS3 cells, through increased recruitment in response to CA3 input, may be responsible for OLM cell silencing during sharp wave-associated ripples (SWRs; Katona et al., 2014). In other words, VGCs in the proximal dendrites of IS3 cells might facilitate the transient SWR recruitment of IS3 cells. In terms of input onto the passive distal dendrites (i.e., in SLM) of IS3 cells in these models, we predict that either stronger input (e.g., simultaneous spatially distributed presynaptic spikes or high-frequency presynaptic input) or adjusted synaptic parameters (e.g., rise time or decay time) would be necessary to recruit IS3 cells to fire a somatic spike, since we found that input from a single presynaptic spike was inefficient in doing so.

This may be similar to CA1 pyramidal cells, which exhibit denser innervation through the perforant path relative to SC inputs (Kajiwara et al., 2008). In addition, in CA1 pyramidal cells, perforant path inputs evoked AMPA-mediated EPSCs with longer rise and decay times than SC inputs (Otmakhova et al., 2002). Although the amplitudes of AMPA-mediated EPSCs from both inputs are not statistically different, the longer perforant path-evoked time course suggests a higher likelihood of synaptic summation. This also raises the possibility that the activation of distal inputs might trigger local cooperative mechanisms that would enhance the influence of SLM synapses on somatic spiking. Perforant path inputs on distal dendrites of CA1 pyramidal cells can initiate local Ca^2+^ spikes through NMDAR- and voltage-gated calcium channel-mediated Ca^2+^ influx (Golding et al., 2002). It is conceivable that a functionally similar mechanism could exist in IS3 cells, albeit one that would likely involve a different combination of Ca^2+^ sources, given the heterogeneity of local Ca^2+^ mechanisms in CA1 interneurons (Camiré and Topolnik, 2012). Furthermore, the input from the entorhinal cortex generates theta rhythmic sinks coupled with theta rhythmic sources in the CA1 SLM and SLM/SR border (Kamondi et al., 1998). Since IS3 cells exhibit intrinsic theta oscillations and are capable of synchronizing OLM cells at theta frequencies (Tyan et al., 2014), they are well positioned to contribute to the generation of these SLM/SR rhythmic patterns.

### Other model database approaches: similarities and differences

In comparison with fully automated approaches using other brute-force techniques (Prinz et al., 2003; Günay et al., 2008; Sekulić et al., 2014), evolutionary algorithm multi-objective optimization techniques (Druckmann et al., 2007, 2013; Hay et al., 2011) or control theoretical approaches (Brookings et al., 2014), the semi-automated strategy has a somewhat different goal. Rather than a focus only on conductance values of the different VGCs in the model populations, it aims to also suggest what VGCs need to be present to match given electrophysiological features in cell types whose intrinsic properties lack any characterization. As such, we felt that retaining elements of hand tuning as well as lumping experimental data around chosen features (Fig. 3) were reasonable at this stage. Moving forward, our subsequently developed models with experimental confirmation of VGC types could then be used as reference models in fully automated approaches with more sophisticated algorithmic techniques.

Brookings et al. (2014) offer a control theoretical approach that focuses on temporal alignment with experimental data and is not feature based, unlike the approach used here. Hay et al. (2011) possessed experimental backpropagating action potential-evoked calcium spike recordings from proximal and distal dendrites in neocortical layer 5 pyramidal cells to constrain their model explorations. While this is feasible to obtain for pyramidal cells, dendritic recordings in many interneuron types are much more challenging, due to the smaller diameters of interneuron dendrites and the heterogeneity of interneuron types. For this reason, we relied on dendritic Ca^2+^ imaging data, which can be indicative of action potential propagation based on previous works (CA1 pyramidal cells: Spruston et al., 1995; Golding et al., 2001; CA1 OLM cells: Martina et al., 2000; Topolnik et al., 2009; CA1 basket cells: Hu et al., 2010; Camiré and Topolnik, 2014).

As well, the approach used by Hay et al. (2011) relies on a base knowledge of layer 5 pyramidal cell channel types and conductance ranges, which has not yet been obtained for IS3 cells. Similarly, to generate a large database of OLM cell models necessary to explore conductance densities and channel distributions, Sekulić et al. (2014) based their models on experimental data for CA1 OLM cell channels and conductance ranges obtained from previous work before using a high-performance computing cluster. In this sense, our approach is useful for focusing on intrinsic properties that are unknown and are of potential theoretical and functional importance.

Although it is unclear whether or not the IS3 features chosen for the models to capture are sufficient, in previous work from Druckmann et al. (2011), it was found that simple current steps were appropriate stimuli to reveal the dynamics of the cell. Here we chose representative IS3 features from the experimental data that we wanted to capture, and particular current step sizes were chosen to encompass each of the four chosen features (Table 2). Our CIP choices were based on the experimental data and features in hand, and with these choices we aimed to obtain representative models. As we were not aiming for optimal models per se, we did not focus on examining different CIPs to see what might be ideal, but rather to make choices that were encompassing of the data. Also, our approach did not focus on automating the capturing of the variability in the experimental data (Druckmann et al., 2007; Brookings et al., 2014), but rather used non-automated, flexible hand tuning to ensure that model feature measurements fell within the distributions found experimentally.

### Limitations

The major morphological limitation is whether or not axonal branches are important in this scenario (M1 vs M2 morphology). From one perspective, including this feature is computationally expensive and requires additional assumptions regarding axonal biophysical properties. From another perspective, excluding it can limit the uses of the model in future projects. Also it seems clear that a large proportion of IS3 surface area is occupied by axonal arborization (i.e., 65% in this case), which means that there is a fairly large loss in surface area once axonal branches are removed. Importantly though, once the passive parameters are optimized in both morphologies, the inclusion of somatic channels has similar effects (data not shown) on the action potential shape and timing measurements in both the M1 and M2 morphologies. Therefore, we can assume that adjusting the passive properties in the remaining axon segments is enough to counteract the effects of surface area loss without largely altering the spiking properties of the cell.

Since our models are minimalistic in regard to only having components that are needed to reasonably capture experimental output, several channels that are likely to be present in IS3 cells were not included. This is namely L-type calcium (*I*_CaL_) and hyperpolarization-activated cyclic nucleotide-gated (HCN; *I*_h_) channels. For one, *I*_h_ seems to be present due to observations of channel-specific effects on the experimental voltage recordings (i.e., hyperpolarization sag). Second, our preliminary Ca^2+^ imaging data show that *I*_CaL_ makes a small contribution to bAP-CaTs in proximal dendrites. Additionally, a previous study (Vinet and Sík, 2006) has also indicated that calretinin-positive cells in the CA1 area of the hippocampus express L-type calcium subunits in small proportion, as well as T-type, N-type, R-type, and P-type calcium channels in larger proportions. Although we examined simulations with some of these channels using our approach as well as with only hand tuning, we did not observe any marked improvements in the measurements of the model, in comparison with the experimental data. In other words, model quality is usually maximized when conductance values for these channels are very small, regardless of the channel distribution or assortment of channel types. It is to be noted that these channel models were based on a previous OLM cell model (Lawrence et al., 2006). The involvement of these channels should, however, be investigated in larger database simulations (Sekulić et al., 2014) since they may likely play subtler roles in governing IS3 electrophysiology and dendritic Ca^2+^ dynamics. For example, although the inclusion of a small HCN channel conductance in the model would drastically improve the hyperpolarization regime measurements, too high of a conductance would detrimentally affect all of the depolarization regime measurements. Finding an appropriate conductance parameter space for HCN channels might be possible using higher-resolution database searches with inclusion of calcium VGC types. With more detailed parameter searches, it may also be possible to ameliorate the measurements in which the SDprox.1 and SDprox.2 databases had little overlap with the experimental data (e.g., spike rate, interspike interval, spike threshold).

Since our models include only minimal types of VGCs required, it might be premature to make direct comparisons with conductance values reported for other cell types. Nevertheless, when considering the estimates of channel conductance using a fast dynamic-clamp technique in CA1 oriens/alveus interneurons (Lien and Jonas, 2003), it was found that the addition of perisomatic Kv3 conductance [maximum conductance (*G*_max_) = 140 nS] with an increased deactivation rate could generate irregular firing patterns with dampened action potential amplitudes, similar to what is seen in IS3 cells. In our approach, however, we found that optimal conductance ranges for S.2 fast delayed rectifier potassium channels were between 0.25 and 1 S/cm^2^. Assuming a spherical soma with a radius of 10 μm and a minimal conductance of 0.25 S/cm^2^, we obtain the following conductance estimate:

$$G_{\text{max}}=\left(\text{conductance})\;\times (\text{Somatic Surace Area}\right)=\left(0.25\;S/{\text{cm}}^{2})\;\times \;(4\pi r^{2}\right)=\left(0.25\times 10^{6}\mu S/{\text{cm}}^{2})\times (4\pi {\left(0.001\;\text{cm}\right)}^{2}\right)=\pi \;\mu S=3142\;nS\text{.}$$

Although this is only an approximation of *G*_max_ (i.e., at least 22 times larger than that in the study by Lien and Jonas, 2003), it points to a likely consequence of having a very minimal set of active properties (i.e., two types of inward currents and two types of outward currents in this case). We expect that with the addition of other channel types, the fast delayed rectifier potassium conductance values of the model will undergo a “balanced decrease” to be able to continue capturing IS3 cell features.

It is also worth mentioning that many assumptions regarding bAP amplitudes were derived from the bAP-CaT amplitudes, while the observation of bAP-CaTs implies the activation of calcium channels along with sodium and potassium channels, and, thus, may depend on the spatial distribution of Ca^2+^ channels. However, for the purposes of this analysis, we have assumed that bAP-CaTs reflect the bAP spatial profile in at least the proximal dendrites. Notably, previous studies have shown that dendritic Ca^2+^ imaging can be highly predictive of AP propagation in dendrites in other hippocampal cell types (CA1 pyramidal cells: Spruston et al., 1995; Golding et al., 2001; CA1 OLM Cells: Martina et al., 2000; Topolnik et al., 2009; CA1 basket cells: Hu et al., 2010; Camiré and Topolnik, 2014).

Finally, in our IS3 cell model, we incorporated a relatively simple model representation of stochastic gating (i.e., using Gaussian white noise), compared with more realistic models of stochastic gating (Fox, 1997; Dorval, 2006; Goldwyn et al., 2011). Despite this, since we know from previous work that Gaussian white noise, in combination with specific VGC types, is sufficient to elicit both irregular firing (Stiefel et al., 2013) as well as subthreshold spectral properties (Morin et al., 2010; Yoshida et al., 2011; Sritharan and Skinner, 2012), this minimal representation seems reasonable. Also, in the absence of particular biological details of stochastic gating in IS3 cells, a more detailed representation is not warranted.

### Concluding remarks and future studies

As mentioned, it is possible to use our developed models as base reference models for larger-scale model database approaches to overcome some of the limitations when searching for appropriate parameter values (Sekulić et al., 2014) in the absence of detailed and larger sets of experimental recordings.

The developed models can also be used to consider functional contributions of IS3 cells by providing theoretical predictions on their recruitment and microcircuit interactions, keeping in mind the above limitations.

Although IS3 cells have been shown to exhibit both irregular and regular adaptive firing, the conditions required for either of these are unknown. It will therefore be necessary for experimentalists to investigate the functional ratios of excitatory and inhibitory inputs that control IS3 cell firing. 

Furthermore, by modeling different layer-specific synaptic inputs to the developed IS3 model, we can predict what types of inputs can drive firing patterns similar to those observed in IS3 cells during electrophysiological recordings. Ultimately, we aim to use these multi-compartment models to help understand the functional contribution of these cells to network oscillations such as theta rhythms.

## Synthesis

The decision was a result of the Reviewing Editor Leonard Maler and the peer reviewers coming together and discussing their recommendations until a consensus was reached. A fact-based synthesis statement explaining their decision and outlining what is needed to prepare a revision is listed below. The following reviewer(s) agreed to reveal their identity: Astrid Prinz

The reviewers both agree that this Ms is potentially worthy of publication in eNeuro. While the first reviewer has only minor criticisms, the second reviewer has asked for numerous substantial revisions to be made.

I do agree with the suggestions made by the second reviewer. Please revise the manuscript in accordance with the reviewer comments. The revised Ms will then be re-reviewed by the second reviewer and the Editor.

The full reviews are included below.

Reviewer #1

I believe the manuscript moderately advances the field by combining advantages of different (manual and automated) approaches to neuronal model parameter tuning in a semi-automated hybrid algorithm that might be generalizable to other models at a high level. I am saying "moderate" and "high level" because in my opinion the particular steps chosen by the authors, and which steps to automate and which ones to perform manually, seem relatively idiosyncratic choices to the particular cell type and modeling objectives. Nonetheless, the manuscript presents an example for a hybrid approach that, in individually modified form, might be useful for other researchers.

Overall this manuscript is interesting and the work appears to be well motivated, executed, and discussed. I have only some minor and very minor comments:

MINOR:

Lines 263, 264: The equation given for the normalized Euclidean distance does not contain any normalization term. Since different measures i have different units, this renders the sum meaningless - it would be adding apples to oranges. I suspect this is simply a typo. The original equation for the normalized Euclidean distance in the cited source (Gunay et al. 2009) divides each term i under the sum by the standard deviation of the respective measure. Please correct the equation if that is what you did. If not, please justify how this is a "normalized" distance. Because the distance measure is so important in the optimization algorithm, this is a minor but crucial revision to make to the manuscript.

When Figure 3 is first referred to in the manuscript (around line 284), the terms S.1, S.2, SD, etc. for the different conductance distribution scenarios have not been introduced yet. I therefore found the Figure impossible to understand and that point. The terms are finally introduced around line 462 and I now see what's going on in the figure, but this should come much earlier.

The parametrizations of the membrane currents used in the models are given in Table 4 by providing literature references and genealogy numbers and the basic mathematical structure of the dynamical equations, but exact parameters are not provided. As such, anybody trying to know details or reproduce the work will have to refer to the literature anyway to get the parameters. So I suggest to either 1) provide only the literature reference and a brief description of the current (name, ion type, inactivation/non-inactivating, fast/slow, low-threshold/high-threshold), or 2) go all the way and provide reference, equation, AND parameters. My preference would be 2), although that would add yet another table to the manuscript.

Throughout: Many of the fonts used in the figures and tables are illegible because they are so tiny. Extreme examples are axis labels in Figure 4, and some of the equations in Table 4. Especially in Table 4, this could easily be solved by introducing more line breaks between equations, which would make much better use of all the empty spaces in columns 2 - 4.

VERY MINOR:

Line 136: I believe the denominator on the left-hand side of the equation should be F_0, same as on the right-hand side.

Line 291 and 292 state that the precise conductance values in the parameter space plots (dimensional stacks) can be extrapolated from the scale bars on the x and y axes. I don't see how this can be achieved, because lines 232 and 233 state that 3-4 parameter values that encompass the hand-tuned values were chosen for each parameter, but what these values were is not stated anywhere (unless I missed it). Maybe an additional table could take care of that.

Line 472: "continuous from" should be "continuous with".

Line 713: delete "with".

Line 786: change "minimal weights" to "minimal synaptic weights".

Line 828: change "statistically indifferent" to "not statistically different".

Please provide scale bars in Figure 1A.

Model voltage traces throughout (starting with Figure 3A): I suggest cutting out a lot of the time between the four current injections. This will allow for the injections (which are the interesting part here) to be stretched out in time. That way it may actually become possible to see individual spikes and perhaps even their shapes.

Reviewer #2

Automated model fitting is a difficult and pressing issue in the field, and will become imperative if we ever want to create simulations such as the blue brain in a time efficient way. This particular paper is not very clear or user-friendly in its methodology (very much to the detriment of the paper), but its ideas are fundamentally valid and interesting.

The paper is confusing, long winded and at times very opaque. I am not sure how they chose search parameters and initial values beyond "it seemed to work".

The paper at hand, "Using 'Cyborg' To Develop Multi-Compartment Models That Predict Biophysical Properties Of Interneuron Specific 3 (IS3) Cells In Hippocampus" presents a method for fitting detailed multi-compartmental neuron models to experimental data, looking specifically at IS3 hippocampal interneurons. The method, dubbed 'cyborg' (by randomly choosing letters from a more descriptive long-hand name) consists of choosing a hand-tuned baseline model, that is sequentially refined by a set of (partially hand-tuned and partially automated) protocols (features) and measurements. The basic idea of using identical protocols and measuring the Euclidean distance between model output and experimental data to quantify model performance, and iteratively modifying the kinetics of ionic conductances, removing or adding currents, to finally obtain a decent fit to data, is intriguing. Further, it is satisfying to see that the authors are able to experimentally verify the predicted dendritic distributions of KDR and KA currents. However the draft fall short on several fronts. First off, the writing is very technical, exhaustively wordy and long winded, and yet it still doesn't explain crucial details in the correct order. Additionally, it fails to mention several important papers that have previously addressed similar problems (see citations below, 1,2,3). More importantly though, the manuscript at hand presents itself as a "methods" paper, when it is really about a scientific result, aided by a semi-automated, and semi-novel method just like many other scientific publications. This is to say that while the results can stand on their own, I am not sure that the methods are readily reproducible or user-friendly and as such the paper does not stand on its now quite yet. In summary, the paper at hand is generally publication-worthy but needs a thorough streamlining, and potential re-orientation of its focus.

General Comments:

There is previous work using fully automated approach for determining channel density, using multi-objective optimisation (1,2,3, below). This is worth comparing and discussing.

Regarding the protocols (CIPs) used: Are they general, or just for your particular cell type? I think it would be helpful to elaborate on how to choose appropriate protocols for a particular model/expt at hand, especially in the light of this being about a method. Even if you choose to focus on the result itself, it would be helpful to discuss the choices of stimuli because the protocol choices, feature choices, and elimination choices appear to be hand tuned for the specific case. How general are they?

Additionally, there seems to be no way of assessing if the CIPs and features and measurements are sufficient to capture all of the essential behaviour. Did you try other protocols that proved to be useless, i.e. did you calculate the normalized distance using another protocol that wasn't used in the fitting, and saw that your method also brings the fit closer to this protocol.

It seems that a lot of important steps in your method require hand-tuning, and expert information about the system at hand. For example, you start with a baseline model, and determine which currents to use, determine the shape of the distribution of currents. Later you replacing currents, seemingly at random. The only automated process is to run the models over a few different parameter values, computing the resulting features, and then comparing with data. Is it really necessary to present the paper as a general automated method like you did? More importantly, does this qualify as a novel method with a cute name? It seems much more honest to say that you fit some parameters of a previously uncharacterized neuron, and built a nice model to capture its behavior.

Minor comments:

Cyborg is a somewhat unnecessary / glitzy / cute-isa acronym that falls short of its promise. While I understand your choice in using it, I would encourage a refocus on the experimental result, i.e. "building multi-compartmental models of IS3 cells in hippocampus that predict biophysical properties"

intro line 51: please define more clearly what you mean by 'population modelling'

intro line 53: using the word 'database' here is a bit uninformative. What exactly does it mean?

intro lines 65-66: you describe the method of Sekuli'c et al. 2014, and say that it requires one to have a starting reference model, and then generates model databases to be compared to specific experimental data. Then you describe the method you present here. It appears to be exactly the same, or very similar. Please explain more clearly how your model relates, and/or builds on this previous work. Without this, it is not a very clear motivation for your method, nor an extension of the previous work.

Methods line 225 and onward: It would be helpful if you could provide a short title for each step which states what it is about. You only do this for step 0 (set up stage).

Methods line 231-242: You describe the CIPs here, in step 1, even though you say that this is part of step 0. Maybe you should say all of this in step 0. If I am correct, it's not part of step 1 which is automated, while the choice of features and protocols is hand selected.

Methods lines 225-301: maybe think about including a flowchart as a figure to illustrate the steps of cyborg.

Methods: What are the parameters and what values do you use? You never say clearly what the parameters are, and you don't seem to give values for the parameters in the text, nor any rationale for what parameter values you picked. Is this also hand-tuned? I see that parameter ranges are given in table 6, but this is not mentioned here, nor motivated anywhere.

Methods line 274: You assume that each of the four features is equally important, but what about each measurement inside each feature? they may have different scales and thus should also be normalized before combining.

Results 383-385: You mention that you chose four features, but really, table 2 shows that there are several 'measurements' for each one of these. e.g., depolarization with normal spiking contains 10 items, such as inter-spike interval, first spike time, etc. I think this is confusing. You could call these 'protocols' instead of features. The features are the 'measurements' that you get from the protocols later. IN any case, it should be clarified.

Results line 395 and figure 2 dashed lines: Please motivate how you choose representative values? Right now it seems random, and not automated. Also, do you choose the values independent of one another, or did you choose values based on one particular trace?

Results lines 450-460: Please say clearly what the parameters are in each of the cases.

Results line 477 and figure 3B: You do not state what the actual parameter values are that you use anywhere, except in figure 6, in which there are only ranges given. also, you only plot 3 choices for each one. How do you know that this is sufficient?

References:

1) Druckmann S1, Hill S, Schürmann F, Markram H, Segev I., A hierarchical structure of cortical interneuron electrical diversity revealed by automated statistical analysis. Cereb Cortex. 2013 Dec;23(12):2994-3006. doi: 10.1093/cercor/bhs290. Epub 2012 Sep 17.

2) Druckmann S1, Banitt Y, Gidon A, Schürmann F, Markram H, Segev I.

Front Neurosci. 2007 Oct 15;1(1):7-18. doi: 10.3389/neuro.01.1.1.001.2007. eCollection 2007.

A novel multiple objective optimization framework for constraining conductance-based neuron models by experimental data.

3) Automatic parameter estimation of multicompartmental neuron models via minimisation of trace error with control adjustment

Ted Brookings, Marie L. Goeritz and Eve Marder, J Neurophysiol 112:2332-2348, 2014. First published 9 July 2014; doi:10.1152/jn.00007.201

## References

[B1] Acsády L, Arabadzisz D, Freund TF (1996a) Correlated morphological and neurochemical features identify different subsets of vasoactive intestinal polypeptide-immunoreactive interneurons in rat hippocampus. Neuroscience 73:299–315. 8783251 10.1016/0306-4522(95)00610-9

[B2] Acsády L, Görcs TJ, Freund TF (1996b) Different populations of vasoactive intestinal polypeptide-immunoreactive interneurons are specialized to control pyramidal cells or interneurons in the hippocampus. Neuroscience 73:317–334. 8783252 10.1016/0306-4522(95)00609-5

[B3] Brookings T, Goeritz ML, Marder E (2014) Automatic parameter estimation of multicompartmental neuron models via minimization of trace error with control adjustment. J Neurophysiol 112:2332–2348. 10.1152/jn.00007.2014 25008414 PMC4274917

[B4] Cai X, Liang CW, Muralidharan S, Kao JP, Tang CM, Thompson SM (2004) Unique roles of SK and Kv4.2 potassium channels in dendritic integration. Neuron 44:351–364. 10.1016/j.neuron.2004.09.026 15473972

[B5] Camiré O, Topolnik L (2012) Functional compartmentalisation and regulation of postsynaptic Ca2+ transients in inhibitory interneurons. Cell Calcium 52:339–346. 10.1016/j.ceca.2012.05.001 22656961

[B6] Camiré O, Topolnik L (2014) Dendritic calcium nonlinearities switch the direction of synaptic plasticity in fast-spiking interneurons. J Neurosci 34:3864–3877. 10.1523/JNEUROSCI.2253-13.2014 24623765 PMC6705275

[B7] Carnevale NT, Hines ML (2006) The neuron book. Cambridge, UK: Cambridge UP.

[B8] Chamberland S, Salesse C, Topolnik D, Topolnik L (2010) Synapse-specific inhibitory control of hippocampal feedback inhibitory circuit. Front Cell Neurosci 4:130. doi:10.3389/fncel.2010.00130. 10.3389/fncel.2010.00130 21060720 PMC2972748

[B9] Chapman CA, Lacaille JC (1999) Intrinsic theta-frequency membrane potential oscillations in hippocampal CA1 interneurons of stratum lacunosum-moleculare. J Neurophysiol 81:1296–1307. 10085356 10.1152/jn.1999.81.3.1296

[B10] Chitwood RA, Hubbard A, Jaffe DB (1999) Passive electrotonic properties of rat hippocampal CA3 interneurones. J Physiol 515:743–756. 10.1111/j.1469-7793.1999.743ab.x 10066901 PMC2269181

[B11] Coetzee WA, Amarillo Y, Chiu J, Chow A, Lau D, McCormack T, Moreno H, Nadal MS, Ozaita A, Pountney D, Saganich M, Vega-Saenz de Miera E, Rudy B (1999) Molecular diversity of K+ channels. Ann N Y Acad Sci 868:233–285. 10414301 10.1111/j.1749-6632.1999.tb11293.x

[B12] Dayan P, Abbott LF (2005) Chapter 5. Model neurons I: neuroelectronics In: Theoretical neuroscience: computational and mathematical modeling of neural systems. Cambridge, MA: MIT.

[B13] De Schutter E, Bower JM (1994) An active membrane model of the cerebellar Purkinje cell. I. Simulation of current clamps in slice. J Neurophysiol 71:375–400. 7512629 10.1152/jn.1994.71.1.375

[B14] Dorval AD (2006) The rhythmic consequences of ion channel stochasticity. Neuroscientist 12:442–448. 10.1177/1073858406290793 16957006

[B15] Druckmann S, Banitt Y, Gidon A, Schürmann F, Markram H, Segev I (2007) A novel multiple objective optimization framework for constraining conductance-based neuron models by experimental data. Front Neurosci 1:7–18. 10.3389/neuro.01.1.1.001.2007 18982116 PMC2570085

[B16] Druckmann S, Berger TK, Schürmann F, Hill S, Markram H, Segev I (2011) Effective stimuli for constructing reliable neuron models. PLoS Comput Biol 7:e1002133. 10.1371/journal.pcbi.1002133 21876663 PMC3158041

[B17] Druckmann S, Hill S, Schürmann F, Markram H, Segev I (2013) A hierarchical structure of cortical interneuron electrical diversity revealed by automated statistical analysis. Cereb Cortex 23:2994–3006. 10.1093/cercor/bhs290 22989582

[B18] Fox RF (1997) Stochastic versions of the Hodgkin-Huxley equations. Biophys J 72:2068–2074. 10.1016/S0006-3495(97)78850-7 9129809 PMC1184401

[B19] Francavilla R, Luo X, Magnin E, Tyan L, Topolnik L (2015) Coordination of dendritic inhibition through local disinhibitory circuits. Front Synaptic Neurosci 7:5. 10.3389/fnsyn.2015.00005 25767448 PMC4341546

[B20] Gentet LJ, Stuart GJ, Clements JD (2000) Direct measurement of specific membrane capacitance in neurons. Biophys J 79:p314–p320. 10.1016/S0006-3495(00)76293-X 10866957 PMC1300935

[B21] Golding NL, Kath WL, Spruston N (2001) Dichotomy of action-potential backpropagation in CA1 pyramidal neuron dendrites. J Neurophysiol 86:2998–3010. 11731556 10.1152/jn.2001.86.6.2998

[B22] Golding NL, Staff NP, Spruston N (2002) Dendritic spikes as a mechanism for cooperative long-term potentiation. Nature 418:326–331. 10.1038/nature00854 12124625

[B23] Goldwyn JH, Imennov NS, Famulare M, Shea-Brown E (2011) Stochastic differential equation models for ion channel noise in Hodgkin-Huxley neurons. Phys Rev E Stat Nonlin Soft Matter Phys 83:041908. 10.1103/PhysRevE.83.041908 PMC3279159 21599202

[B24] Gulyás AI, Hájos N, Freund TF (1996) Interneurons containing calretinin are specialized to control other interneurons in the rat hippocampus. J Neurosci 16:3397–3411. 8627375 10.1523/JNEUROSCI.16-10-03397.1996 PMC6579144

[B25] Günay C, Edgerton JR, Jaeger D (2008) Channel density distributions explain spiking variability in the globus pallidus: a combined physiology and computer simulation database approach. J Neurosci 28:7476–7491. 10.1523/JNEUROSCI.4198-07.2008 18650326 PMC5771640

[B26] Günay C, Edgerton JR, Li S, Sangrey T, Prinz AA, Jaeger D (2009) Database analysis of simulated and recorded electrophysiological datasets with PANDORA's toolbox. Neuroinformatics 7:93–111. 10.1007/s12021-009-9048-z 19475520 PMC2786174

[B27] Hay E, Hill S, Schürmann F, Markram H, Segev I (2011) Models of neocortical layer 5b pyramidal cells capturing a wide range of dendritic and perisomatic active properties. PLoS Comput Biol 7:e1002107. 10.1371/journal.pcbi.1002107 21829333 PMC3145650

[B28] Hernández-Pineda R, Chow A, Amarillo Y, Moreno H, Saganich M, Vega-Saenz de Miera EC, Hernández-Cruz A, Rudy B (1999) Kv3.1-Kv3.2 channels underlie a high-voltage-activating component of the delayed rectifier K+ current in projecting neurons from the globus pallidus. J Neurophysiol 82:1512–1528. 10482766 10.1152/jn.1999.82.3.1512

[B29] Hines ML, Morse T, Migliore M, Carnevale NT, Shepherd GM (2004) ModelDB: a database to support computational neuroscience. J Comput Neurosci 17:7–11. 10.1023/B:JCNS.0000023869.22017.2e 15218350 PMC3732827

[B30] Holmes WR (2010) Passive cable modeling In: Computational modeling methods for neuroscientists (De Schutter E, ed), pp 233–358. Cambridge, MA: MIT.

[B31] Hu H, Martina M, Jonas P (2010) Dendritic mechanisms underlying rapid synaptic activation of fast-spiking hippocampal interneurons. Science 327:52–58. 10.1126/science.1177876 19965717

[B32] Kajiwara R, Wouterlood FG, Sah A, Boekel AJ, Baks-te Bulte LT, Witter MP (2008) Convergence of entorhinal and CA3 inputs onto pyramidal neurons and interneurons in hippocampal area CA1–an anatomical study in the rat. Hippocampus 18:266–280. 10.1002/hipo.20385 18000818

[B33] Kamondi A, Acsády L, Wang XJ, Buzsáki G (1998) Theta oscillations in somata and dendrites of hippocampal pyramidal cells in vivo: activity-dependent phase-precession of action potentials. Hippocampus 8:244–261. 10.1002/(SICI)1098-1063(1998)8:3&amp;lt;244::AID-HIPO7&amp;gt;3.0.CO;2-J 9662139

[B34] Katona L, Lapray D, Viney TJ, Oulhaj A, Borhegyi Z, Micklem BR, Klausberger T, Somogyi P (2014) Sleep and movement differentiates actions of two types of somatostatin-expressing GABAergic interneuron in rat hippocampus. Neuron 82:872–886. 10.1016/j.neuron.2014.04.007 24794095 PMC4041064

[B35] Lawrence JJ, Saraga F, Churchill JF, Statland JM, Travis KE, Skinner FK, McBain CJ (2006) Somatodendritic Kv7/KCNQ/M channels control interspike interval in hippocampal interneurons. J Neurosci 26:12325–12338. 10.1523/JNEUROSCI.3521-06.2006 17122058 PMC6675427

[B36] Leão RN, Mikulovic S, Leão KE, Munguba H, Gezelius H, Enjin A, Patra K, Eriksson A, Loew LM, Tort AB, Kullander K (2012) OLM interneurons differentially modulate CA3 and entorhinal inputs to hippocampal CA1 neurons. Nat Neurosci 15:1524–1530. 10.1038/nn.3235 23042082 PMC3483451

[B37] Lein ES, Hawrylycz MJ, Ao N, Ayres M, Bensinger A, Bernard A, Boe AF, Boguski MS, Brockway KS, Byrnes EJ, Chen L, Chen L, Chen TM, Chin MC, Chong J, Crook BE, Czaplinska A, Dang CN, Datta S, Dee NR, (2007) Genome-wide atlas of gene expression in the adult mouse brain. Nature 445:168–176. 10.3389/fncel.2010.00130 17151600

[B38] Lien CC, Jonas P (2003) Kv3 potassium conductance is necessary and kinetically optimized for high-frequency action potential generation in hippocampal interneurons. J Neurosci 23:2058–2068. 12657664 10.1523/JNEUROSCI.23-06-02058.2003 PMC6742035

[B39] Lien CC, Martina M, Schultz JH, Ehmke H, Jonas P (2002) Gating, modulation and subunit composition of voltage-gated K(+) channels in dendritic inhibitory interneurones of rat hippocampus. J Physiol 538:405–419. 11790809 10.1113/jphysiol.2001.013066 PMC2290075

[B40] Maccaferri G, McBain CJ (1995) Passive propagation of LTD to stratum oriens-alveus inhibitory neurons modulates the temporoammonic input to the hippocampal CA1 region. Neuron 15:137–145. 7619518 10.1016/0896-6273(95)90071-3

[B41] Magee JC (2000) Dendritic integration of excitatory synaptic input. Nat Rev Neurosci 1:181–190. 10.1038/35044552 11257906

[B42] Magee J, Hoffman D, Colbert C, Johnston D (1998) Electrical and calcium signaling in dendrites of hippocampal pyramidal neurons. Annu Rev Physiol 60:327–346. 10.1146/annurev.physiol.60.1.327 9558467

[B43] Martina M, Vida I, Jonas P (2000) Distal initiation and active propagation of action potentials in interneuron dendrites. Science 287:295–300. 10634782 10.1126/science.287.5451.295

[B44] Migliore M, Cook EP, Jaffe DB, Turner DA, Johnston D (1995) Computer simulations of morphologically reconstructed CA3 hippocampal neurons. J Neurophysiol 73:1157–1168. 7608762 10.1152/jn.1995.73.3.1157

[B45] Migliore M, Hoffman DA, Magee JC, Johnston D (1999) Role of an A-type K+ conductance in the back-propagation of action potentials in the dendrites of hippocampal pyramidal neurons. J Comput Neurosci 7:5–15. 10481998 10.1023/a:1008906225285

[B46] Morin F, Haufler D, Skinner FK, Lacaille JC (2010) Characterization of voltage-gated K+ currents contributing to subthreshold membrane potential oscillations in hippocampal CA1 interneurons. J Neurophysiol 103:3472–3489. 10.1152/jn.00848.2009 20393060

[B47] Otmakhova NA, Otmakhov N, Lisman JE (2002) Pathway-specific properties of AMPA and NMDA-mediated transmission in CA1 hippocampal pyramidal cells. J Neurosci 22:1199–1207. 11850447 10.1523/JNEUROSCI.22-04-01199.2002 PMC6757557

[B48] Prinz AA, Billimoria CP, Marder E (2003) Alternative to hand-tuning conductance-based models: construction and analysis of databases of model neurons. J Neurophysiol 90:3998–4015. 10.1152/jn.00641.2003 12944532

[B49] Saraga F, Wu CP, Zhang L, Skinner FK (2003) Active dendrites and spike propagation in multi-compartment models of oriens-lacunosum/moleculare hippocampal interneurons. J Physiol 552:673–689. 10.1113/jphysiol.2003.046177 12923216 PMC2343469

[B50] Sekulić V, Lawrence JJ, Skinner FK (2014) Using multi-compartment ensemble modeling as an investigative tool of spatially distributed biophysical balances: application to hippocampal oriens-lacunosum/moleculare (O-LM) cells. PLoS One 9:e106567. 10.1371/journal.pone.0106567 25360752 PMC4215854

[B51] Sivagnanam S, Majumdar A, Yoshimoto K, Astakhov V, Bandrowski A, Martone ME, Carnevale NT (2013). Introducing the Neuroscience Gateway . Paper presented at IWSG 2013, Zürich, Switzerland, June.

[B52] Spruston N, Schiller Y, Stuart G, Sakmann B (1995) Activity-dependent action potential invasion and calcium influx into hippocampal CA1 dendrites. Science 268:297–300. 7716524 10.1126/science.7716524

[B53] Sritharan D, Skinner FK (2012) Fluctuating inhibitory inputs promote reliable spiking at theta frequencies in hippocampal interneurons. Front Comput Neurosci 6:30. doi:10.3389/fncom.2012.00030. 10.3389/fncom.2012.00030 22654751 PMC3359426

[B54] Stiefel KM, Englitz B, Sejnowski TJ (2013) Origin of intrinsic irregular firing in cortical interneurons. Proc Natl Acad Sci U S A 110:7886–7891. 10.1073/pnas.1305219110 23610409 PMC3651468

[B55] Taylor AL, Hickey TJ, Prinz AA, Marder E (2006) Structure and visualization of high-dimensional conductance spaces. J Neurophysiol 96:891–905. 10.1152/jn.00367.2006 16687617

[B56] Tigerholm J, Migliore M, Fransén E (2013) Integration of synchronous synaptic input in CA1 pyramidal neuron depends on spatial and temporal distributions of the input. Hippocampus 23:87–99. 10.1002/hipo.22061 22996230

[B57] Topolnik L (2012) Dendritic calcium mechanisms and long-term potentiation in cortical inhibitory interneurons. Eur J Neurosci 35:496–506. 10.1111/j.1460-9568.2011.07988.x 22304664

[B58] Topolnik L, Chamberland S, Pelletier JG, Ran I, Lacaille JC (2009) Activity-dependent compartmentalized regulation of dendritic Ca2^+^ signaling in hippocampal interneurons. J Neurosci 29:4658–4663. 10.1523/JNEUROSCI.0493-09.2009 19357290 PMC6665741

[B59] Tóth K (2010) Glutamatergic neurotransmission in the hippocampus In: Hippocampal microcircuits (Cutsuridis V, Graham BP, Cobb S, Vida I, eds), pp 107–117). New York: Springer.

[B60] Tyan L, Chamberland S, Magnin E, Camiré O, Francavilla R, David LS, Deisseroth K, Topolnik L (2014) Dendritic inhibition provided by interneuron-specific cells controls the firing rate and timing of the hippocampal feedback inhibitory circuitry. J Neurosci 34:4534–4547. 10.1523/JNEUROSCI.3813-13.2014 24671999 PMC6608127

[B61] Uebachs M, Opitz T, Royeck M, Dickhof G, Horstmann MT, Isom LL, Beck H (2010) Efficacy loss of the anticonvulsant carbamazepine in mice lacking sodium channel β subunits via paradoxical effects on persistent sodium currents. J Neurosci 30:8489–8501. 10.1523/JNEUROSCI.1534-10.2010 20573896 PMC6634624

[B62] Vinet J, Sík A (2006) Expression pattern of voltage-dependent calcium channel subunits in hippocampal inhibitory neurons in mice. Neuroscience 143:189–212. 10.1016/j.neuroscience.2006.07.019 16938402

[B63] Yang S, Tang CM, Yang S (2015) The shaping of two distinct dendritic spikes by a-type voltage-gated K(+) channels. Front Cell Neurosci 9:469. doi: 10.3389/fncel.2015.00469. 10.3389/fncel.2015.00469 26696828 PMC4673864

[B64] Yoshida M, Giocomo LM, Boardman I, Hasselmo ME (2011) Frequency of subthreshold oscillations at different membrane potential voltages in neurons at different anatomical positions on the dorsoventral axis in the rat medial entorhinal cortex. J Neurosci 31:12683–12694. 10.1523/JNEUROSCI.1654-11.2011 21880929 PMC3177240

